# Genome-enhanced detection and identification of fungal pathogens responsible for pine and poplar rust diseases

**DOI:** 10.1371/journal.pone.0210952

**Published:** 2019-02-06

**Authors:** Marie-Josée Bergeron, Nicolas Feau, Don Stewart, Philippe Tanguay, Richard C. Hamelin

**Affiliations:** 1 Laurentian Forestry Centre, Canadian Forest Service, Natural Resources Canada, Québec, Québec, Canada; 2 Forest Sciences Centre, Department of Forest and Conservation Sciences, University of British Columbia, Vancouver, British Columbia, Canada; 3 Institut de Biologie Intégrative des Systèmes, Université Laval, Québec, Québec, Canada; University of Helsinki, FINLAND

## Abstract

Biosurveillance is a proactive approach that may help to limit the spread of invasive fungal pathogens of trees, such as rust fungi which have caused some of the world’s most damaging diseases of pines and poplars. Most of these fungi have a complex life cycle, with up to five spore stages, which is completed on two different hosts. They have a biotrophic lifestyle and may be propagated by asymptomatic plant material, complicating their detection and identification. A bioinformatics approach, based on whole genome comparison, was used to identify genome regions that are unique to the white pine blister rust fungus, *Cronartium ribicola*, the poplar leaf rust fungi *Melampsora medusae* and *Melampsora larici-populina* or to members of either the *Cronartium* and *Melampsora* genera. Species- and genus-specific real-time PCR assays, targeting these unique regions, were designed with the aim of detecting each of these five taxonomic groups. In total, twelve assays were developed and tested over a wide range of samples, including different spore types, different infected plant parts on the pycnio-aecial or uredinio-telial host, and captured insect vectors. One hundred percent detection accuracy was achieved for the three targeted species and two genera with either a single assay or a combination of two assays. This proof of concept experiment on pine and poplar leaf rust fungi demonstrates that the genome-enhanced detection and identification approach can be translated into effective real-time PCR assays to monitor tree fungal pathogens.

## Introduction

Invasive fungal pathogens of trees represent a global threat to natural forests and tree plantations, which may result in large economic losses and, in some cases, changes to ecosystem functions and a reduction in biodiversity [1–3]. The worldwide spread of invasive species is directly related to anthropogenic activities and the number of new invasive pathogens within a country can be correlated to its economic activity, as measured by its gross domestic product [1, 4]. Prevention, which is supported by biosurveillance, is the most efficient approach in slowing introduction and spread of invasive alien pathogens [5]. However, biosurveillance of plant pathogens is challenging. Some oomycete and fungal pathogens can live on plant material that is asymptomatic and can go undetected during visual inspections. Others can be misidentified given the paucity of discriminant morphological characters in some groups of fungi and the great taxonomic diversity encountered during surveys and inspections. These factors increase the probability of failure to detect alien pathogens which may lead to their establishment and eventual outbreaks.

These challenges are particularly pressing in rust fungi which can cause some of the most devastating diseases of pines, poplars and eucalyptus [6–10]. Heteroecious rust fungi alternate on both susceptible pycnio-aecial and uredinio-telial hosts, further complicating their detection and identification, particularly when a plant is host to more than one rust species occurring in sympatry. For example, *Larix* spp. may be infected by both *Melampsora* and *Melampsoridium* species, which can cause poplar, willow and birch leaf rusts on their uredinio-telial hosts [11, 12].

The white pine blister rust, caused by the fungus *Cronartium ribicola* J. C. Fisch. (Basidiomycota, Pucciniales), is one of the major factors that contributed to the decline of the North American populations of five needle pines. This exotic pathogen was accidentally introduced to Canada and USA on infected white pine seedlings imported from Europe and intended for reforestation following extensive logging in the 19th century. It has since established and spread in most areas where its pycnio-aecial (*Pinus* subsection *Strobus*) and uredinio-telial (*Ribes* in the Grossulariaceae or less commonly *Pedicularis* and *Castilleja* in the Orobanchaceae) hosts cohabit [6]. *Melampsora medusae* Thüm. f. sp. *deltoidae* Shain and *Melampsora larici-populina* Kleb. (Basidiomycota, Pucciniales) cause poplar leaf rust, leading to severe damage in commercial poplar plantations. *Melampsora medusae* f. sp. *deltoidae* is native to North America, where it alternates between its pycnio-aecial (*Larix* spp., *Pseudotsuga* spp. and young *Pinus* spp.) and uredinio-telial (*Populus* spp. and hybrids in sections *Aigeiros* and *Tacamahaca*) hosts. It has spread throughout most of the world where poplars are grown and it has been classified as a quarantine pest in Europe [13, 14]. *Melampsora larici-populina* is an invasive Eurasian fungus that was first reported in Western and Eastern North America in 1991 and 2002, respectively, where it has been able to overwinter and infect some hybrid poplars which had never been affected by poplar leaf rust before [15, 16].

During the last decade, efforts have been made to improve the methods of detection of these important pathogens, using molecular tools. For example, PCR assays, based on single nucleotide polymorphisms located within the nuclear ribosomal internal transcribed spacer (ITS) region, were developed for the detection of *Melampsora* species (*M*. *medusae* f. sp. *deltoidae*, *M*. *larici-populina* and *M*. *allii-populina*) that attack cultivated poplars belonging to sections *Aigeiros* and *Tacamahaca* [17]. Real-time PCR assays were also developed for the simultaneous detection of both *formae speciales* of *M*. *medusae*, based on polymorphisms found within the ITS region, and also for the detection of the *forma specialis deltoidae* only, based on the large ribosomal RNA subunit [18, 19]. Regions other than ribosomal DNA were also targeted. For example, two distinct single-copy genes, MS208 and MS277, were used in a duplex real-time assay, to discriminate *M*. *larici-populina* and *M*. *allii-populina*, respectively [20]. However, due to the low number of polymorphic DNA regions currently identified and suitable for the design of discriminant assays, other means needed to be explored.

An approach that uses whole genome comparisons to identify genes that are unique to targeted species as well as shared within a group of related species is showing promise to address this issue [21, 22]. This approach has the advantage of reducing the risk of false positive or false negative assignment and producing robust assays that are not dependent upon single nucleotide polymorphisms. We present here the use of the GEDI (genome-enhanced detection and identification) approach [21] to design specific real-time assays targeting *C*. *ribicola*, *M*. *medusae* and *M*. *larici-populina* and taxa belonging to *Cronartium* and *Melampsora* genera and evaluate their performance for the detection of pine and poplar leaf rust pathogens from environmental samples.

## Material and methods

### Isolate collection, monosporal culture and DNA isolation

A worldwide collection of samples from target and non-target species among the Pucciniales was established, with the assistance of collaborators or international herbaria (S1–S4 Tables). No collection permits were required for samples collected from Canadian Crown land. In the cases where samples were collected from private plantations, permission was obtained from the owners. For international samples, collaborators obtained permission. No sampling of endangered species was conducted. Most of the samples were DNA barcoded to confirm their species identification or the presence of pathogens on environmental material. Mono-uredinial cultures of *Cronartium ribicola* and *Melampsora* spp. were also used, which were obtained as follows: an initial inoculation of a detached blackcurrant or poplar leaf was made with urediospores sampled from a single uredium observed on environmental material. After 8–10 days post-inoculation, urediospores sampled from one of the uredia developed on the detached leaf were inoculated on a fresh detached leaf. This step was repeated one more time before propagation of the inoculum on multiple detached leaves was initiated.

DNA was isolated using a phenol-chloroform extraction method, the DNeasy Plant Mini procedure (Qiagen GmbH, Hilden, Germany) or protocols implemented in the laboratories of collaborators who provided us with DNA samples.

To estimate the sensitivity of the real-time PCR assays, spore suspensions of *Cronartium ribicola*, *Melampsora medusae* f. sp. *deltoidae* and *Melampsora larici-populina* were prepared by adding absolute ethanol to bulks of dessicated spores and filtering using cell strainers (100 μm Nylon; BD Falcon, USA). Uniform spore suspension was maintained by vortex mixing and serial dilutions (1:10) were prepared with absolute ethanol plus the surfactant SilWet L-77 (1μL/10mL of ethanol; Momentive Performance Materials Inc., USA). Spore concentration of these serially diluted suspensions was estimated with a hemacytometer, under a light microscope at 250X magnification, to enable the transfer of 1.5 X 10^6^, 2.5 X 10^4^, 5 X 10^3^, 1.25 X 10^3^, 5 X 10^2^, 50 and 25 spores to 2 mL Safe-Lock tubes (Eppendorf AG, Hamburg, Germany), in three biological replicates. Ethanol was evaporated using a CentriVap concentrator (Labconco Corp., Kansas City, MO) and spores were stored at -20°C until DNA isolation. The QIAamp DNA Micro kit (Qiagen GmbH, Hilden, Germany) was used for DNA isolation, as described in the *Isolation of Genomic DNA from Tissue Samples* section of the manufacturer’s handbook (04/2003 version). Briefly, spores resuspended in 180 μL of Buffer ATL and 1 μL of reagent DX (Qiagen GmbH) were mechanically disrupted twice with an acid-washed tungsten carbide bead (3 mm, Qiagen GmbH), using a Mixer Mill 300 (Qiagen GmbH, Haan, Germany) run at 30 beats per sec for 1.5 min. The lysis was carried out overnight in a heating block at 56°C, in the presence of Proteinase K (20 μL of >600 mAU/mL solution). For the first two hours of the lysis, the samples were vortexed for 10 sec every 10 min. To enhance binding of DNA to the QIAamp membrane, carrier RNA was added to Buffer AL. Following the binding and washing steps, elution was carried out with 50 μL of Buffer EB (Qiagen), except for DNA isolated from 25 and 50 spores, for which 25 μL of Buffer EB was applied onto the column. DNA LoBind tubes (Eppendorf AG) were used as collection tubes at the elution step. The QIAamp DNA Micro kit was also used for DNA isolation from single telia of *C*. *ribicola*, following the same described method.

### Identification of unique genes based on comparative genomics

Identification of gene and genome regions suitable for developing highly specific real-time PCR assays for *M*. *larici-populina*, *M*. *medusa*e, *C*. *ribicola* and the *Melampsora* and *Cronartium* genera was carried out using the GEDI method described in a previous study [21]. Briefly, seventeen rust genomes were considered for analyses, including three *Cronartium*/*Endocronartium* and six *Melampsora* species obtained previously by pair-end Illumina sequencing (Canada’s Michael Smith Genome Sciences Centre, Vancouver, Canada) [21]. The 122,475 protein models predicted from these *de novo* assemblies were combined with those obtained from the genomes of *M*. *larici-populina* [23], *Cronartium quercuum* f. sp. *fusiforme*, *Puccinia graminis* f. sp. *tritici* [23], *P*. *triticina*, *P*. *striiformis*, *Mixia osmundae* [24], *Sporobolomyces roseus* and *Rhodotorula graminis* [25]. Protein sets were submitted to a clustering process with the orthoMCL algorithm (BLASTp e-value cutoff of 1e-05, minimum similarity cutoff of 50%, inflation parameter of 1.5) [26]. Among the 90,135 clusters found, “singlets” i.e. clusters containing putative unique protein models to single species *M*. *larici-populina*, *M*. *medusae* f. sp. *deltoidae* or *C*. *ribicola* were identified (S1A Fig) and submitted to a false positive filtering by running a TBLASTn (1e-20) against the other rust genomes included in the study and a BLASTp (1e-20) on NCBI-nr. Putative genus-specific protein models to *Melampsora* and *Cronartium* were identified by finding clusters of orthologs/paralogs shared among species within the genus but not with any other rust species (S1B Fig). Filtering of the genus-specific clusters for false positives was carried out by running TBLASTn (1e-20) against rust genomes from alternative genera. For all five targeted taxonomic groups, clusters with positive hits were discarded and the remaining gene sequences were retrieved for each of the protein models. They were submitted to Primer3 V4.0.0 [27] for PCR primer and probe design with the following parameters: amplicon size of 100 to 150 bp; primer optimal size of 20 bases (18–27 bases) with an optimal Tm of 60°C (58–62°C); probe optimal size of 20 bases (18–27 bases) with an optimal Tm of 65°C (64–68°C).

### Real-time PCR primer and probe design

Among the hundreds of sequence cluster files (Cluster_n.fas) generated by our bioinformatics method for each targeted taxon, up to twenty clusters were selected per taxon, based on the homology spanned over the entire length of sequences (n ≥ 2) forming ortholog/paralog clusters. Oligo Analyzer V1.5 (Gene Link, http://www.genelink.com/tools/gl-oe.asp) was used to improve the primer/probe combinations initially generated by our bioinformatics method. For an optimal amplification at 60°C, the following rules were applied whenever possible: 1) Tm values for primers between 58–60°C and 8–10 °C higher for probes; 2) minimum primer length of 16 bases; 3) probe length between 21–27 bases; 4) no G at the extreme 5’ end of the probe; 5) no probes with four or more identical nucleotides, especially Gs; and 6) secondary structure and 3’ self-complementarity primer-dimer formation avoided or limited. The parameters used for Tm calculation were: salt concentration of 50 mM and DNA concentration of 250 pM. Furthermore, primers (MEL40, MEL100 and MEL176 assays) and probe (MEL176 assay) with degenerate bases were designed due to interspecific variation that was observed within the alignment of targeted region sequences representative of five to six *Melampsora* spp.

### Experimental conditions of PCR

Following design optimization, primer pairs were synthesized (Integrated DNA Technologies Inc., Coralville, IA, USA) and tested against a reduced panel of DNA samples from target and non-target species among the Pucciniales to select those that specifically amplified the targeted taxonomic groups (refer to footnotes *a* and *b* in S1 Table and footnotes *a*, *b* and *c* in S2 Table). One μL of purified DNA solution was used as template for the PCR amplification using 1.0 U of Platinum Taq DNA polymerase (Invitrogen, Life Technologies, Carlsbad, CA, USA), in a 25 μL reaction mix comprising 1X PCR buffer minus Mg, 1.6 mM of MgCl_2_, 200 μM of dNTPs and 1 μM of each primer. Thermocycling conditions included an initial heat activation step of 3 min at 94°C followed by 35 cycles of denaturation at 92°C for 30 sec, primer annealing at 60°C for 30 sec and extension at 72°C for 1 min. A final extension step at 72°C for 10 min completed the program. DNA fragment analysis was performed with capillary electrophoresis using the QIAxcel Advanced system (Qiagen GmbH, Hilden, Germany). Products of specific amplification were purified on glass microfiber UniFilter (GE Healthcare Whatman) and sequenced (Sanger’s method) at the Genome sequencing and genotyping platform of the CHUL medical research centre (Québec, QC, Canada). Sequences were edited using Sequencher V4.8 (Gene Codes Corporation, Ann Arbor, MI, USA) and deposited in GenBank: MH171727-MH171903.

Primer pairs that resulted in the specific amplification of targeted taxonomic groups were evaluated in combination with TaqMan probes (Tables 1 and 2) on a larger panel of DNA samples (all samples displayed in S1 and S2 Tables). Real-time PCR was performed in a 10 μL reaction mix comprising 1X QuantiTect Multiplex PCR NoRox Master Mix (Qiagen, Valencia, CA), 0.6 μM of each primer, 0.1 μM of double-quenched TaqMan probe (5’FAM/ZEN/3’IB-FQ; Integrated DNA Technologies Inc.) and 1 μL of template DNA, using the 7500 Fast Real-Time PCR system (Applied Biosystems, Life Technologies, Foster City, CA). Thermocycling conditions included an initial heat activation step of 15 min at 95°C followed by 50 cycles of denaturation at 95°C for 15 sec and primer annealing/extension at 60°C for 1 min. Fluorescence was read at the end of each extension step.

**Table 1:** Genus- and species-specific assays for hierarchical detection of *Cronartium* spp. and *Cronartium ribicola*.

Assay	Targeted taxon	Primer or TaqMan probe^a^	Sequence (5’ → 3’)	Tm (NN)^b^	Amplicon length	GenBank accession no.
CRO30	*Cronartium* spp.	Cronartium30F	ATCGTTCTCATCGTCAGCG	57.1 °C	104 bp	MH171752-MH171772
		Cronartium30R	TTAAAGCTTTGAAGGCGACG	56.0 °C		
		Cronartium30T (47–70)	TCTTCACTGCCGTCTACTTTCTGC	64.2 °C		
CRO46	*Cronartium* spp.	Cronartium46F	CCGGTTATGGCGATCTCAC	60.4 °C	117 bp	MH171773-MH171780
		Cronartium46R	AAATCGGGCACTCTGTTCTG	58.9 °C		
		Cronartium46T (72–98)	ATCGCTTTCGATGGACGTGATCATGGC	67.4 °C		
CRIB65	*Cronartium ribicola*	Crib65Fm	CTTTTGCTACTTGCGCATTTGA	57.0 °C	102 bp	MH171727-MH171734
		Crib65Rm	CTCTGGTGAGGAGCTATCG	60.1 °C		
		Crib65T (61–83)	CTACAGGGTTCACCTGCTTCGCC	68.7 °C		
CRIB146	*Cronartium ribicola*	Crib146F	TCAGGTTTCGGATTTTGAGG	55.8 °C	115 bp	MH171735-MH171742
		Crib146R	TGCTTCTGAGGCTTTTTGGT	58.5 °C		
		Crib146T (37–63)	CATACCTGATACGAGTGGACTGAATGA	63.6 °C		
CRIB190	*Cronartium ribicola*	Crib190Fm	CTCCAGCTACAGTGGGTA	59.9 °C	121 bp	MH171743-MH171751
		Crib190Rm	CCTTGTCTGTTGGTGAGGT	59.2 °C		
		Crib190T (50–77)	ACATGGGAACGACAAGGACAATTTGGAC	66.0 °C		

^a^ In parentheses: probe position within amplicon.

^b^ Values estimated by Oligo Analyzer V1.5.

**Table 2:** Genus- and species-specific assays for hierarchical detection of *Melampsora* spp., *Melampsora medusae* and *Melampsora larici-populina*.

Assay	Targeted taxon	Primer or TaqMan probe^a^	Sequence (5’ → 3’)	Tm (NN)^b^	Amplicon length	GenBank accession no.
MEL40	*Melampsora* spp.	Melampsora40Fm	CCTGGTACTCCAACTATCATCTTA	60.3 °C	131 bp	MH171821-MH171846
		Melampsora40Rm	GAAWGTGCACGCGATTGACG	59.3 °C		
		Melampsora40T (40–66)	CCTCTGAGTGATGGCGAAGACCCTTTA	68.1 °C		
MEL100	*Melampsora* spp.	Melampsora100Fm	CACGAAAGTCBCAAGTGGC	60.6 °C	148 bp	MH171847-MH171875
		Melampsora100Rm	GTRCAGTCATGAGGTACGATA	59.3 °C		
		Melampsora100T (66–85)	CCTCCTCCTCCGCTCTACGC	68.8 °C		
MEL176	*Melampsora* spp.	Melampsora176Fm	GCCCTTGCCGTTGCTAT	60.7 °C	112 bp	MH171876-MH171903
		Melampsora176Rm	GRCTCGTGCTGATCAGTC	60.2 °C		
		Melampsora176Td (37–62)	ACCACTCACCATCCGTAYATCAACGC	68.2 °C		
MM53	*Melampsora medusae*	Mm53F	ACAACCAGGTGACGGAAATC	59.1 °C	129 bp	MH171799-MH171810
		Mm53R	GAATCGTCCGAGGAGTCATT	58.1 °C		
		Mm53Trev (52–28)	CTTAGGTCGTTCACCCTCTGATTCG	64.6 °C		
MM74	*Melampsora medusae*	Mm74Fm	CACCATGCAAATCACCAATCAC	58.4 °C	118 bp	MH171811-MH171820
		Mm74R	TTTGGCTCAGCCTCAGTTTT	58.5 °C		
		Mm74T (29–57)	TGGACCTTCTTTGCAAATCAACACAGTAT	63.4 °C		
MLP104	*Melampsora larici-populina*	Mlp104Fm	CGGCCAGAAATTGTGATGGAT	59.4 °C	104 bp	MH171781-MH171789
		Mlp104R	TGCATAGCCTTTGTGGACAG	59.9 °C		
		Mlp104T(28–56)	CGTATGAACCATTGATTGAAGGCTTGGAC	65.1 °C		
MLP133	*Melampsora larici-populina*	Mlp133Fm	ATGGACCGGGAATATGAAC	56.7 °C	126 bp	MH171790-MH171798
		Mlp133R	TCGTTGATCGTATCGTGGAA	56.0 °C		
		Mlp133T(33–55)	CGCATCAGGTGGATTTGGTGAAG	62.9 °C		

^a^ In parentheses: probe position within amplicon.

^b^ Values estimated by Oligo Analyzer V1.5.

### Specificity of the real-time PCR assays

**Fig 1:**
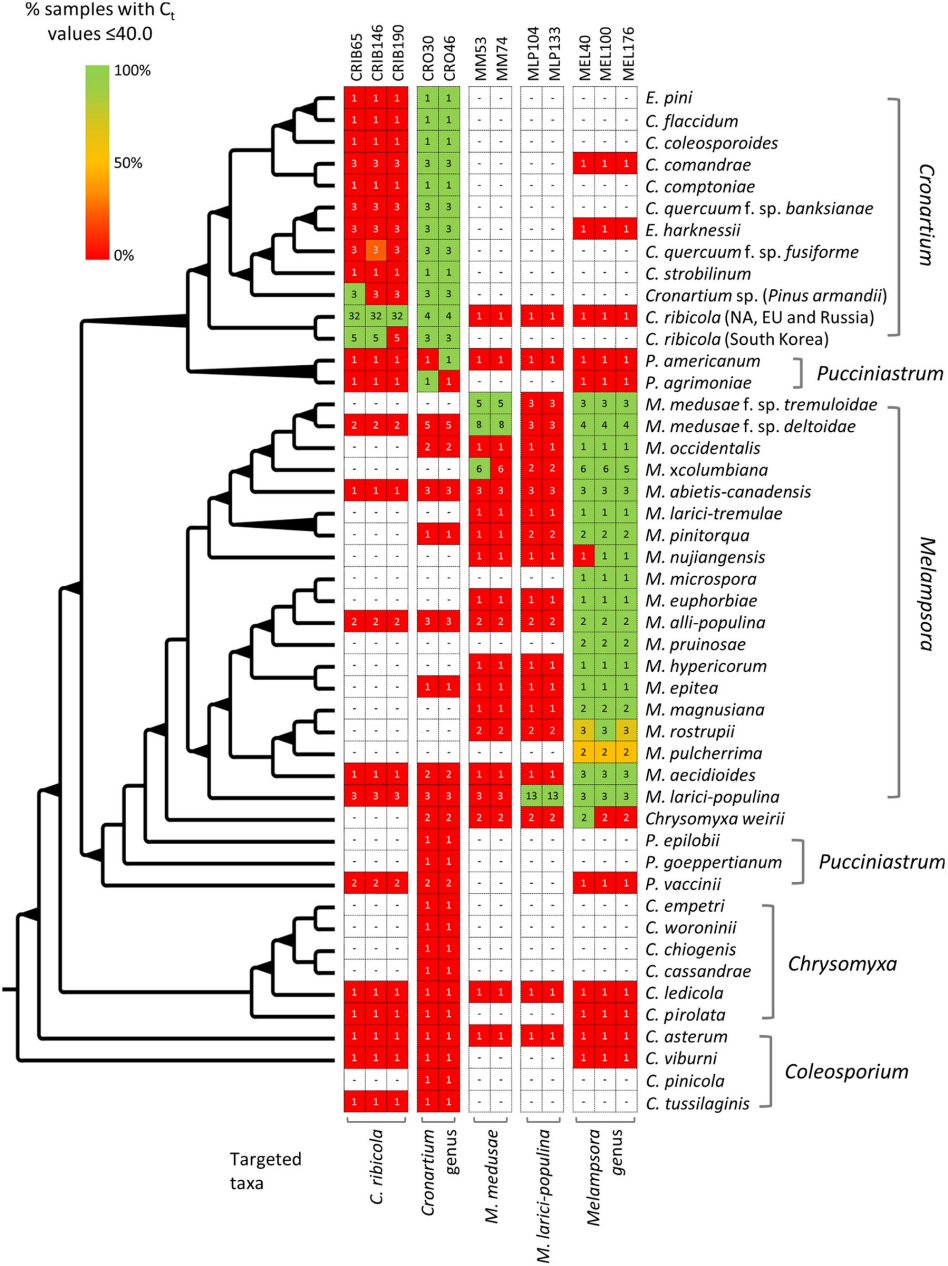
Specificity of the pine and poplar rust assays. The NJ phylogenetic tree represents evolutionary relationships among the rust samples used, inferred from an alignment of ITS sequences; bolded nodes received bootstrap values ≥ 80%. Rows represent the number of rust samples used within each taxon and columns stand for the different assays. For each taxon, the relative abundance of samples with C_t_ values ≤ 40.0, as determined by real-time PCR, is depicted by color scale with the legend at the upper left side.

Mono-uredinial cultures and environmental material from 11 *Cronartium* and 18 *Melampsora* rust species were used to assess the specificity of the assays. Cross-reaction of the assays with DNA from non-target species was also evaluated as well as the performance of the assays in detecting the presence of targeted taxonomic groups on environmental material (Fig 1). For *Cronartium* spp., environmental samples included aeciospores of *C*. *ribicola* produced on naturally infected white pines (*Pinus strobus*), urediospores and single telia produced on blackcurrants (*Ribes nigrum*) and symptomatic white pine stem tissue and chlorotic needle infection spots of white pine seedlings artificially inoculated with *C*. *ribicola*. Insects intercepted in a white pine plantation infected with blister rust were also used as some feed on the nectar of spermogonia produced on the surface of the pine tree bark during the initial spore stage infection on pine [28] (S3 Table). It has been postulated that *Megaselia* spp. and *Paracacoxenus guttatus* are the main insects responsible for cross-fertilization of *C*. *ribicola* [28]. *Megaselia* spp. accounted for seven out of nine insects tested in this study. *Melampsora* spp. environmental samples included aeciospores and urediospores of both *M*. *medusae* f. sp. *deltoidae* and *M*. *larici-populina* produced on naturally infected larch (*Larix laricina*) and poplar (*Populus* spp. and interspecific hybrids), respectively (S4 Table).

### Sensitivity of the real-time PCR assays on a known number of spores

Sensitivity of the real-time PCR assays was determined by testing seven different amounts of spores for *C*. *ribicola*, *M*. *medusae* f. sp. *deltoidae* and *M*. *larici-populina*: 30,000, 500, 100, 25, 10, 2 and 1 spore(s). DNA was isolated from three biological replicates for each amount of spores. Three technical replicates were performed for all reactions, using the same real-time PCR conditions as those described above. Graphs were generated by plotting the number of spores on the x-axis against the mean C_t_ (cycle threshold) values determined by real-time PCR on the y-axis. Each dot represents a biological independent replicate (corresponding to a known amount of spores), which was obtained by averaging the C_t_ values of three technical real-time PCR replicates. Amplification reaction efficiency was calculated from the slope of the lines, using the following formula:

$$E=\;\left(10^{\left(–1/\text{slope}\right)}–\;1\right)\;\times \;100$$

### Data analysis and interpretation

#### Naïve Bayes classifier

We built a naïve Bayes classifier [29] to i. select the optimal combination of real-time PCR assays producing the most accurate detection of the targeted species and ii. interpret assay outcomes. Using a training set of C_t_ values obtained from the collection of positive and negative samples for each targeted taxon (see below), the classifier estimates for each assay the parameters of probability density of the C_t_ values obtained for two given classes X|*i* (positive samples) and X|*j* (negative samples). Then, for any new sample tested with a C_t_ value X, the method computes the posterior probability P(*i*|X) of that sample belonging to the class *i* and the posterior probability P(*j*|X) to belong to *j* according to the naïve Bayes model [29–31]:

$$D\;=\;\frac{P(i|X)}{P(j|X)}\;=\;\frac{\;P(X|i)\;P(i)}{\;P(X|j)\;P(j)}$$

Probability densities *P*(X|*i*) and *P*(X|*j*) have to be integrated into the Bayes model through a function of probability density calculated over the training set. Such a function *f(x)* represents the normal distribution of a numerical attribute x as a function of a mean μ and its standard deviation σ (assuming a normal distribution of C_t_ values in the experiment):

$$f(x)\;=\;\frac{1}{\sqrt{2\pi \sigma }}e^{-\frac{{\left(x-\mu \right)}^{2}}{{2\sigma }^{2}}}$$

with $\mu \;=\;\frac{1}{n}\sum_{i\;=\;1}^{n}x_{i}$ and $\sigma \;=\;\frac{1}{n-1}\sum_{i\;=\;1}^{n}{\left(x_{i}-\mu \right)}^{2}$

Finally, assuming independence between each assay, the Bayes model is applied recursively for each selected assay; thus, it is possible to use every *a posteriori* probability to be i P(i|Xn) as *a priori* probability for the next assay. P(i|Xn) becomes P(i) to calculate P(i|X*n+1*) on the next Bayes iteration as follows:

$$D\;=\;\frac{P(i|X1,\;X2,\ldots \;Xn)}{P(j|X1,\;X2,\ldots \;Xn)}\;=\;\frac{\;P(X1,\;X2,\ldots \;Xn|i)P(i)}{P(X1,\;X2,\ldots \;Xn|j)P(j)}$$

The method then classifies the sample data according to the largest posterior probability (D positive predicts *i* else predicts *j*).

#### Training sets

Training sets of positive and negative samples were built with C_t_ values obtained from the tests made on mono-uredinial cultures and environmental rust samples (See *Specificity of the real-time PCR assays*). Only samples whose species identity was verified by DNA barcoding of the ITS region were retained (S1–S5 Tables). DNA barcodes were obtained from previous studies for *Melampsora* and *Chrysomyxa* spp. [32–34]; for the other rust fungi, the ITS region was amplified/sequenced using ITS-1f [35] and ITS-4 [36] (or ITS-4BR [37] or ITS-4BR2: 5’- GGATTATCACCCTCAATGAT -3’) primers, under the same thermochemical conditions as described above, except for the primer annealing temperature which was set at 54–55°C.

#### Assay combinations

For each targeted taxonomic group, we tested each single assay and all assay combinations with the naïve Bayes classifier to reassign samples of the corresponding training set to be either positive or negative (Python script available upon request). Samples were then identified as being true positive (TP), true negative (TN), false positive (FP) and false negative (FN), depending on the correspondence between their DNA barcode and the result of the naïve Bayes classifier. TP samples were those with a DNA barcode corresponding to the targeted species and identified as being positive with the naïve Bayes classifier; TN were samples with a DNA barcode other than the targeted species and identified as being negative with the naïve Bayes classifier; FP were samples with a DNA barcode other than the targeted species and identified as being positive with the naïve Bayes classifier; finally, FN were samples with a DNA barcode of the targeted species that returned a negative identification with the naïve Bayes classifier. The accuracy of a detection assay is then defined as the rate of correct positive (defined as sensitivity of detection) and correct negative (specificity of detection) identifications:

$$Accuracy\;(\%)\;=\;\frac{\;TP\;+\;TN}{TP+TN+FP+FN}$$

The best minimal assay combination is the one that provides the highest accuracy over the entire reference population and includes the lowest number of assays.

#### Simulation of new samples

To circumvent potential issues with unbalanced sets of positive and negative samples and obtain higher precision on the estimates of TP, TN, FP and FN and accuracy rates, we simulated new sets of samples. For each targeted taxonomic group, 1,000 random datasets of 500 positive and 500 negative samples were simulated and accuracy measurement was re-estimated by using the naïve Bayes classifier on these new datasets (Python script available upon request). For each simulated dataset, C_t_ values for the positive samples were obtained based on the modeling of a normal distribution of the experimental data obtained for positive samples. C_t_ values of the negative samples were considered as the left tail part of a distribution right-truncated at the last cycle of the real-time PCR run (i.e. 40.0) and were modeled using an exponential distribution as suggested in Chandelier *et al*. [38].

## Results and discussion

Tree rust-specific and sensitive real-time PCR assays were developed using the GEDI (genome-enhanced detection and identification) method which identifies genome regions that are unique to targeted taxa or groups of related taxa following whole genome comparisons [21]. These assays were designed for species-specific detection of *Cronartium ribicola*, *Melampsora medusae* and *Melampsora larici-populina* and for the detection of taxa belonging to *Cronartium* and *Melampsora* genera.

### Assay design

The GEDI bioinformatics method, previously described [21] and applied in this study, resulted in 1341, 1542 and 1519 unique genes identified as species-specific to *C*. *ribicola*, *M*. *medusae* f. sp. *deltoidae* and *M*. *larici-populina*, respectively. At the genus level, 34 candidate gene clusters uniquely shared among the four *Cronartium*/*Endocronartium* species analysed were identified as compared to 270 among the seven *Melampsora* species (Table 2 in [21]).

### Assay selection, specificity and sensitivity

#### Pine rusts

Subsets of species- and genus-specific candidate genes were screened by PCR on a reduced panel of DNA samples from target and non-target species among the Pucciniales. Among the 20 primer pairs screened for *C*. *ribicol*a, three yielded amplicons with the ten selected *C*. *ribicola* samples collected in North America, Europe and Russia: CRIB65, CRIB146 and CRIB190 (Table 1 and S1 Table). None of these primer pairs produced amplicons with DNA from other rust species (n = 20 samples representative of ten *Cronartium* spp., other than *C*. *ribicola*, and two *Melampsora* spp.). Once the primer pair screening was completed, TaqMan probes were incorporated into the master mixes and the resulting assays were tested on a larger panel of DNA samples (Fig 1 and S1 Table). The specificity of CRIB65, CRIB146 and CRIB190 assays was confirmed, with the exception of the CRIB65 assay that generated a signal with *Cronartium* sp. collected on *Pinus armandii* in China. Multiple lines of evidence, based on host specialization, spore morphology and protein and DNA analyses indicate that this blister rust pathogen on *P*. *armandii* is different from *C*. *ribicola*, even though this fungus has not been recognized as a distinct taxon yet (for a review see [39]). Similarly, another Asian blister rust pathogen, collected on *Pinus koraiensis* in South Korea was detected with CRIB65 and CRIB146 assays, but not with the CRIB190 assay. Phylogenetic relationships of *C*. *ribicola* samples collected on different telial and aecial host species in Eurasia and North America as well as artificial inoculation studies on different telial host species suggest that this pathogen might have sub-species or ecological races [40].

At a higher taxonomic level, eight *Cronartium*-specific candidate genes were screened by PCR on a reduced panel of DNA samples, including 21 samples representative of 11 *Cronartium*/*Endocronartium* spp. and eight samples representative of as many non-target species among the Pucciniales. Two primer pairs yielded amplicons with the 21 selected *Cronartium/Endocronartium* spp. samples: CRO30 and CRO46, but not with DNA from other rust species (Table 1 and S1 Table). TaqMan probes were incorporated into the master mixes of the two selected primer pairs to design real-time PCR assays that were tested on a larger panel of DNA samples (Fig 1 and S1 Table). The specificity of CRO30 and CRO46 assays was confirmed, with the exception of positive signals for the CRO30 assay on a *Pucciniastrum agrimoniae* sample, and the CRO46 assay on a specimen of *Pucciniastrum americanum*. These two rust species are known to be closely related to the *Cronartium* genus [41], as confirmed in our phylogenetic reconstruction (Fig 1).

**Fig 2:**
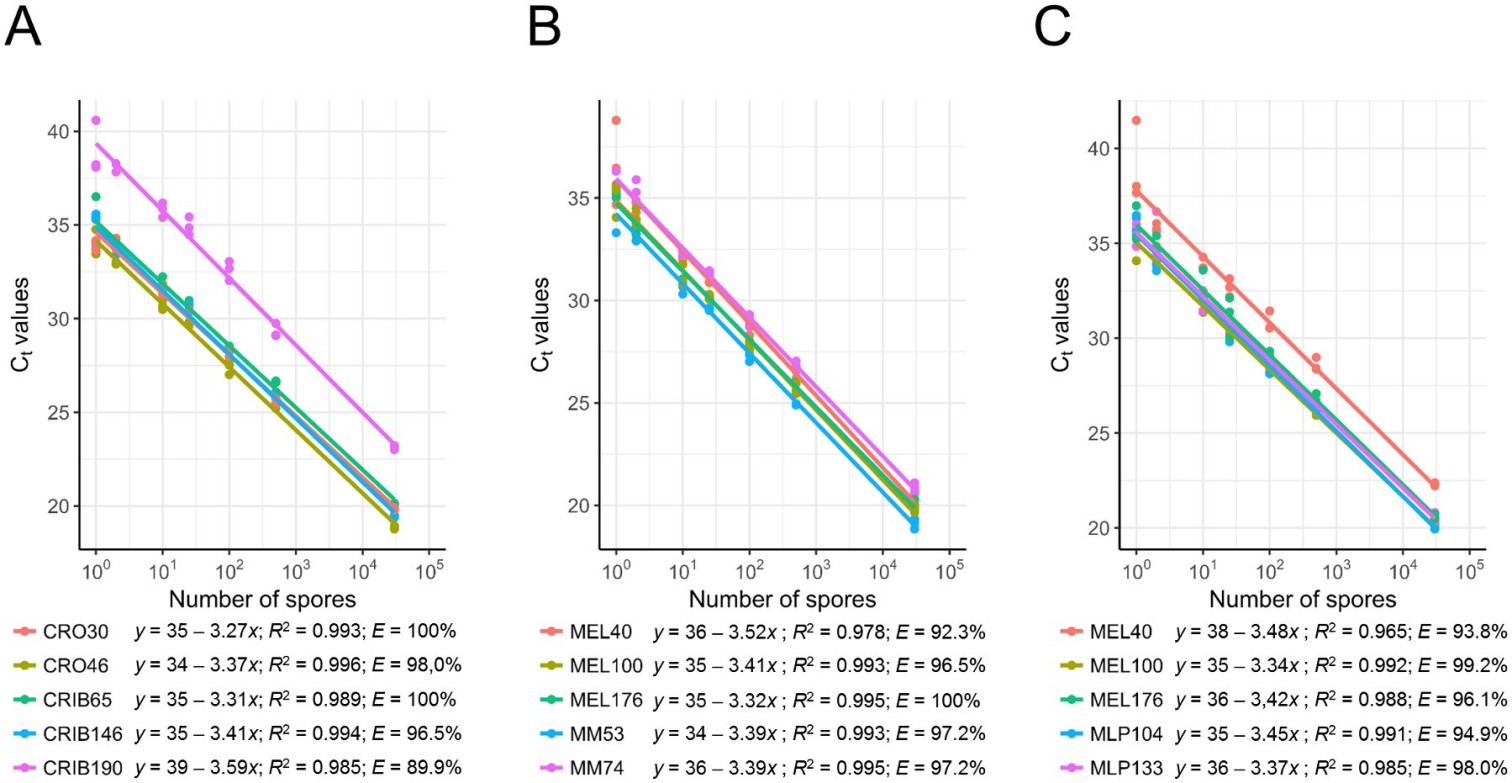
Relationship between the number of *Cronartium ribicola* (A), *Melampsora medusae* f. sp. *deltoidae* (B) and *Melampsora larici-populina* (C) spores from which DNA was isolated and C_t_ values, as determined by real-time PCR. Each dot represents a biological independent replicate (corresponding to a known amount of spores), which was obtained by averaging the C_t_ values of three technical real-time PCR replicates.

Using as few as one spore per reaction resulted in successful detection, as indicated by mean C_t_ values between 33.44 and 40.58, with the five TaqMan assays specific to *C*. *ribicola* or *Cronartium* spp. (S6 Table). Sensitivity in spore detection with the genus-specific assay CRO46 was slightly higher than sensitivity with the other genus-specific assay CRO30 or the species-specific assays CRIB65 and CRIB146 (Fig 2A and S6 Table). The species-specific assay CRIB190 was the least sensitive, with detection at higher C_t_ values for all spore quantities tested (average difference of 3.7 to 4.1 cycles with CRIB65 and CRIB146 assays, respectively). Linearity was observed across the range of spores counted, and from which DNA was isolated, and the mean C_t_ values determined by real-time PCR for each assay (Fig 2A). The high coefficients of determination (*R*^*2*^ = 0.985–0.996) indicated low variability between the three independent DNA isolations performed for each amount of spores of *C*. *ribicola*. Estimates of the efficiency of the amplification reaction were high, with values ranging from 96.5% (CRIB146) to 100% (CRO30 and CRIB65), except for the CRIB190 assay which had an efficiency of 89.9% (Fig 2A).

#### Poplar rusts

Reduced panels of DNA samples from target and non-target species among the Pucciniales were used in PCR screening of subsets of species-specific candidate genes for both *M*. *medusae* f. sp. *deltoidae* and *M*. *larici-populina* and a subset of *Melampsora*-specific candidate genes. Among the ten primer pairs screened for *M*. *medusae* f. sp. *deltoidae*, two yielded amplicons with the eight *M*. *medusae* f. sp. *deltoidae* samples collected in North America and South Africa: MM53 and MM74 (Table 2 and S2 Table). No other rust taxa produced amplicons (n = 20 samples representative of 12 *Melampsora* spp., other than *M*. *medusae* f. sp. *deltoidae*), except for *M*. *medusae* f. sp. *tremuloidae* and *M*. ×*columbiana*. There are two host-specific *formae speciales* within *M*. *medusae*, named *M*. *medusae* f. sp. *deltoidae* (pathogenic on *Populus deltoides* and poplars in section *Aigeiros*) and *M*. *medusae* f. sp. *tremuloidae* (pathogenic on *Populus tremuloides* and poplars in section *Populus*) [42, 43]. A recent phylogenetic study supported the split of these *formae speciales* into two distinct and independent evolutionary lineages i.e. species [33]. Sequencing of the *M*. *medusae* f. sp. *tremuloidae* genome and comparison with the one of *M*. *medusae* f. sp. *deltoidae* should help to identify species-specific genome regions that would allow for the design of real-time PCR assays unique to each of these two rust taxa. The MM53 primer pair produced a faint amplification with some of the *M*. ×*columbiana* samples tested. This result was expected as *M*. ×*columbiana* is an interspecific hybrid between the two North American poplar rusts *M*. *medusae* f. sp. *deltoidae* and *M*. *occidentalis* [44, 45]. When the TaqMan probe was incorporated into the master mix, the resulting MM53 assay generated a signal for all six *M*. ×*columbiana* samples tested. The other assay targeting *M*. *medusae*, MM74, did not generate a signal for any *M*. ×*columbiana* samples tested nor did it generate a signal with the additional species of the expanded panel (Fig 1 and S2 Table). *Melampsora* ×*columbiana* hybrids exhibit various morphological features of both parents, sometimes mixed with the diagnostic alleles of only one of the parental species, indicating different degrees of hybridization from F1 hybrids to back-crosses with one of the parental species [44]. The ability to detect this hybrid with the MM53 and MM74 assays likely depends on the degree of introgression that will determine which of the *M*. *medusae* f. sp. *deltoidae* or *M*. *occidentalis* alleles are present at these two targeted loci in the *M*. ×*columbiana* samples tested.

For *M*. *larici-populina*-specific candidate genes, two out of ten primer pairs screened yielded amplicons with all 11 selected *M*. *larici-populina* samples collected in North America, New Zealand and China: MLP104 and MLP133 (Table 2 and S2 Table). Neither primer pair produced amplicons with DNA isolated from other rust species (n = four samples representative of as many non-target *Melampsora* spp.). Once this primer pair screening was completed, TaqMan probes were incorporated into the master mixes and the resulting assays were tested on a larger panel of DNA samples (Fig 1 and S2 Table). The specificity of the MLP104 and MLP133 assays was confirmed.

At a higher taxonomic level, five *Melampsora*-specific candidate genes were screened by PCR on a reduced panel of DNA samples, including 26 samples representative of 14 *Melampsora* spp. and ten samples representative of as many non-target species among the Pucciniales. Three primer pairs produced amplicons with 24 out of 26 selected *Melampsora* spp. samples: MEL40, MEL100 and MEL176 (Table 2 and S2 Table). No other rust taxa were amplified with these primer pairs. Once the TaqMan probes were incorporated into the master mixes, the resulting assays were tested on the expanded panel. The specificity of the MEL40, MEL100 and MEL176 assays was confirmed, except for the MEL40 assay which generated a signal with the spruce needle rust species *Chrysomyxa weirii* (Fig 1 and S2 Table). This result was not unexpected as *C*. *weirii* is known to share similar teliospore morphology and basidium germination characteristics with representative species of the genus *Melampsora* [46]. Recent multi-gene phylogenies have confirmed *C*. *weirii* as being more closely related to *Melampsora* than the *Chrysomyxa* genus [34], which is corroborated by our phylogenetic reconstruction, based on the ITS region (Fig 1).

Sensitivity of spore detection with the poplar leaf rust assays was similar to that obtained with the pine rust assays, with as few as one spore detected (Fig 2B and 2C and S7 and S8 Tables). Linearity was observed across the range of spores tested, and from which DNA was isolated, and the mean C_t_ values determined by real-time PCR for each assay (Fig 2B and 2C). Low variability was also observed between the three independent DNA isolations performed for each amount of spores of *M*. *medusae* f. sp. *deltoidae* or *M*. *larici-populina* (*R*^*2*^ = 0.965–0.995). Estimates of the efficiency of the amplification reactions were high, with values ranging from 92.3% to 100% (Fig 2B and 2C).

### Tests on environmental samples

#### Pine rusts

DNA obtained from five types of environmental samples was tested with the pine rust assays. Three of the five assays yielded 100% detection success (measured as C_t_ values < 40.0) with inoculated and naturally infected material, regardless of the tissue infected or symptom type (white pine needle and stem tissues, blackcurrant leaves or single telia; S3 Table). One *C*. *ribicola*-specific assay (CRIB190) failed to detect *C*. *ribicola* in three inoculated samples and two naturally infected samples. One *Cronartium*-specific assay (CRO30) failed to detect the pathogen in a single telium sample (S3 Table). When DNA samples associated with C_t_ values > 40.0 were retained, positive results increased to between 90.9% and 100%. Assays CRO30, CRO46, CRIB65 and CRIB146 detected comparable amounts of *C*. *ribicola* DNA from DNA isolated from insects captured in a white pine plantation diseased with blister rust: eight out of the nine isolates produced positive results (88.9%), with C_t_ values < 40.0 (S3 Table). In contrast, positive results decreased to 33.3% with the CRIB190 assay when isolates with C_t_ values > 40.0 were excluded. However, when the C_t_ value threshold was raised to 41.5, all nine DNA isolates produced positive results, which were confirmed by fungal DNA barcoding. Insects associated with spore dispersal might facilitate spermogonia fertilization by carrying *C*. *ribicola* spermatia to the spermogonia, which produce a nectar that attracts the insects. Once fertilization has occurred, a dikaryotic mycelium develops, which later organizes to form aecidia on pines [47].

#### Poplar rusts

All poplar rust assays have the ability to detect *M*. *medusae* f. sp. *deltoidae* and/or *M*. *larici-populina* DNA on naturally infected larch needles and poplar leaves (S4 Table). One hundred percent of DNA isolates from naturally infected larch samples produced positive results with the MEL40, MEL100 and MEL176 assays. The species-specific assays showed that *M*. *medusae* f. sp. *deltoidae* was detected almost four times more frequently than *M*. *larici-populina* on the larch needles analysed. Furthermore, all 33 samples from naturally infected poplar leaves produced positive results with the three genus-specific assays. Co-infection by both *M*. *medusae* f. sp. *deltoidae* and *M*. *larici-populina* was detected in 32 out of the 33 poplar leave samples tested with the species-specific assays. All of these results were corroborated by previous results achieved through real-time PCR assays, using species-specific primers targeting the ITS region [48]. Moreover, the variable abundance, based on C_t_ values of both *Melampsora* species detected on these leaves with our poplar rust assays, targeting single- or low-copy genes, is consistent with observations using an assay targeting the multi-copy region of the internal transcribed spacers 1 and 2 ([49] and unpublished data).

### Accuracy of the assays

Detection of pathogens by real-time PCR usually requires setting a C_t_ value threshold to declare a positive reaction. As originally proposed by Liu and Saint [50], the fluorescent signal of a real-time PCR reaction fits the sigmoidal mathematical model. In theory, under optimal and standardized conditions, the fluorescent threshold value for detecting one copy of the target sequence should be consistent across samples and assays. However, in practice, many factors impact PCR efficiency (e.g. the quantity and quality of the template DNA, the PCR reagents, the thermocycler). As demonstrated by Grosdidier *et al*. [51], specific C_t_ cutoff values need to be calculated for any given target*matrix*thermocycler combination since a single change of target, matrix (physicochemical nature of the reaction mixture) or thermocycler will affect this cutoff value. This approach is empirical and generally based on theoretical, unproven assumptions [51]. Assuming that the C_t_ values obtained from reference collections of positive and negative samples were independent variables [30, 31], we anticipate that the overlap could be interpreted in a Bayesian context. We employed naïve Bayes classifier learning on these distributions to determine the probability that an unknown sample is a true positive. For each real-time PCR assay, the classifier was trained on the C_t_ values obtained on an extensive set of positive and negative samples (mono-uredinial cultures and environmental rust samples) that were previously identified as true positive and true negative samples by using a DNA barcoding approach [32]. Following this learning stage, the classifier assigned unknown samples as being either positive or negative.

We first used the classifier to re-evaluate each sample of the training sets and generate measurements of performance (TP and FP rates, accuracy) for each assay and for each combination of two and three assays. For the three targeted species and the two genera, we obtained an accuracy of detection of 100% (i.e. TP = 100% and FP = 0.0%; Table 3). A single assay was sufficient to reach this level of accuracy for the targets of *C*. *ribicola* with CRIB146, *M*. *larici-populina* with MLP104 or MLP133 and *Cronartium* spp. with CRO46, whereas a minimum of two combined assays were needed for *M*. *medusae* f. sp. *deltoidae* with MM53 + MM74 and *Melampsora* spp. with MEL100 + MEL176 (Table 3). Interestingly, the combination of the two assays CRO30 and CRO46 for detecting *Cronartium* spp. resulted in a lower accuracy than using CRO46 alone; the low accuracy obtained with the assay combination CRO30 + CRO46 likely resulted from the low TP rate (92.7%) and the substantial FP rate (3.5%) yielded by CRO30. This assay failed to detect *C*. *ribicola* on one out of the nine insect vectors, positive with our DNA barcoding approach, as well as one out of 14 single telia harvested on blackcurrant leaves (S3 Table). CRO30 also cross-reacted with *Pucciniastrum agrimoniae* (Fig 1).

**Table 3:** Assignment predictions made with the naïve Bayes classifier.

Pine and poplar rust assays	% TP ^a^	% FP ^a^	Accuracy (%)
*Cronartium* spp.—41/29^b^			
CRO30	92.7 (94.59)	3.5 (11.72)	94.3 (91.44)
**CRO46**	**100.0 (99.11)**	**0.0 (0.45)**	**100.0 (99.33)**
CRO30 + CRO46	100.0 (99.86)	3.5 (2.47)	98.6 (98.70)
*Cronartium ribicola*—64/34^b^			
CRIB65	96.9 (99.43)	5.9 (2.32)	95.9 (98.56)
CRIB146	100.0 (100.0)	0.0 (0.69)	100.0 (99.66)
CRIB190	90.6 (97.95)	0.0 (0.64)	93.9 (98.65)
CRIB65 + CRIB46	100.0 (100.0)	0.0 (0.21)	100.0 (99.90)
CRIB65 + CRIB190	95.3 (99.86)	0.0 (0.11)	96.9 (99.88)
CRIB146 + CRIB190	100.0 (100.0)	0.0 (0.40)	100.0 (99.80)
**CRIB65 + CRIB146 + CRIB190**	**100.0 (100.0)**	**0.0 (0.14)**	**100.0 (99.93)**
*Melampsora* spp.—105/12^b^			
MEL40	92.4 (91.21)	8.3 (7.85)	92.3 (91.68)
MEL100	99.1 (99.61)	0.0 (0.46)	99.2 (99.57)
MEL176	99.1 (99.12)	0.0 (0.23)	99.2 (99.45)
MEL40 + MEL100	99.1 (99.85)	0.0 (0.69)	99.2 (99.58)
MEL40 + MEL176	99.1 (99.77)	0.0 (0.86)	99.2 (99.45)
**MEL100 + MEL176**	**100.0 (99.99)**	**0.0 (0.11)**	**100.0 (99.94)**
MEL40 + MEL100 + MEL176	100.0 (100.0)	0.0 (0.39)	100.0 (99.80)
*Melampsora medusae*—67/31^b^			
MM53	98.5 (99.10)	0.0 (0.39)	99.0 (99.36)
MM74	92.5 (95.04)	3.2 (3.11)	93.9 (95.97)
**MM53 + MM74**	**100.0 (99.93)**	**0.0 (0.88)**	**100.0 (99.52)**
*Melampsora larici-populina*—91/46^b^			
MLP104	100.0 (99.33)	0.0 (0.39)	100.0 (99.47)
MLP133	100.0 (98.90)	0.0 (0.27)	100.0 (99.31)
**MLP104 + MLP133**	**100.0 (99.98)**	**0.0 (0.14)**	**100.0 (99.92)**

Values on the left are estimates obtained from the training set and values on the right, in parentheses, are those obtained from resampling. Lines highlighted in bold characters correspond to the best assay or assay combinations, as predicted after the resampling procedure.

^a^TP: true positive; FP: false positive.

^b^Number of samples tested (positives/negatives).

**Fig 3:**
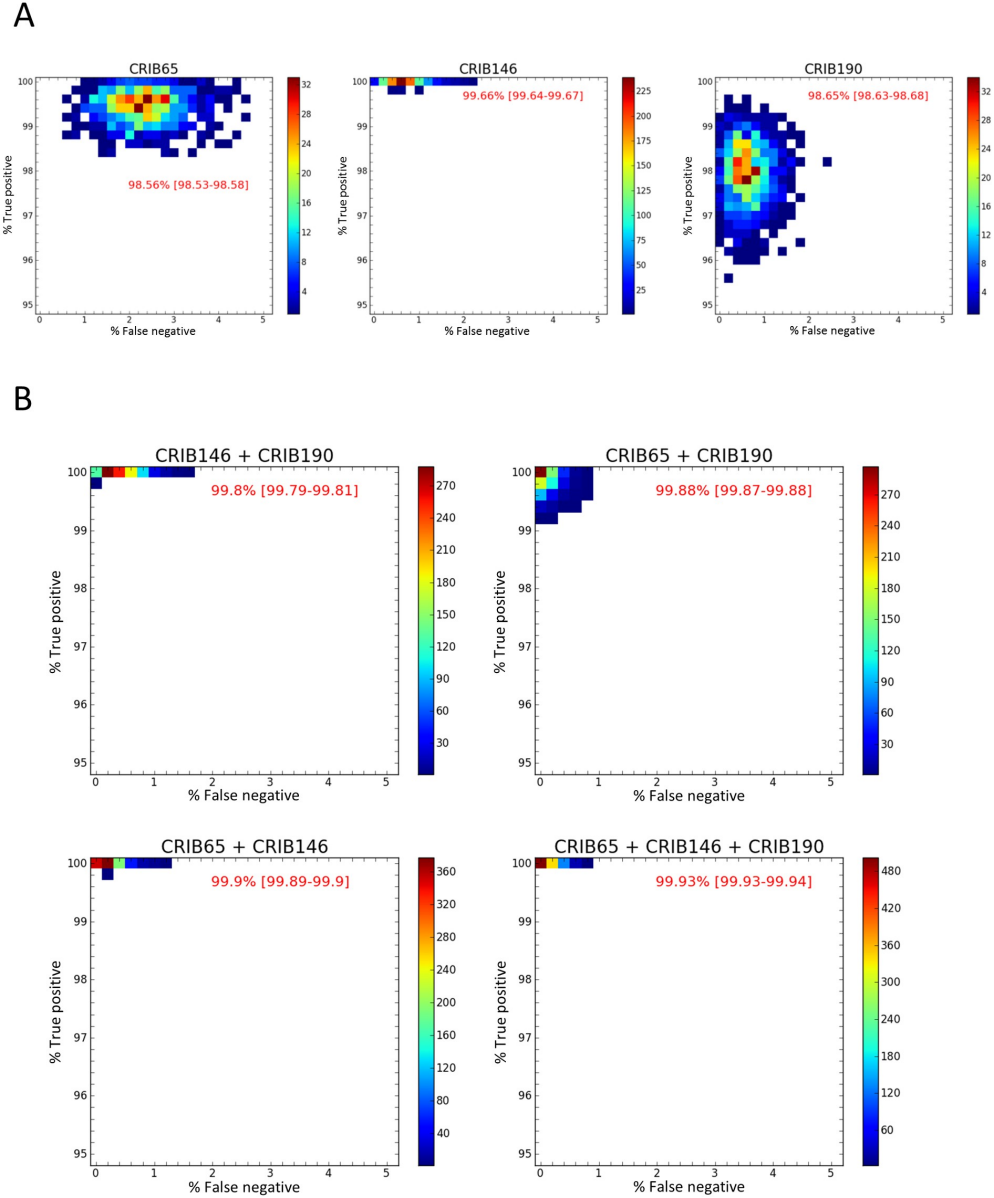
Distribution of true positive (TP) and false positive (FP) rates estimated with a naïve Bayes classifier, over 1,000 simulations of 500 positive and 500 negative samples, for the three specific assays targeting *Cronartium ribicola*. Results are presented for each single assay (A) and for all possible combinations of two or three assays (B). Color scale represents the number of simulations that were assigned into each TP rate X FP rate category. Values in red represent the percentage of accuracy with 95% confidence intervals.

To obtain a higher accuracy, we generated a new dataset of random positive and negative samples for each targeted taxonomic group (500 for each class) based on the C_t_ value distributions of the training sets. Results obtained with the naïve Bayes classifier on the five simulated datasets confirmed those previously obtained for *Cronartium* spp. (highest accuracy for CRO46; 99.33%), *M*. *medusae* f. sp. *deltoidae* (MM53 + MM74; 99.52%) and *Melampsora* spp. (MEL100 + MEL176; 99.94%) (Table 3). In the two other cases (*C*. *ribicola* and *M*. *larici-populina*), analysis of the simulated datasets resulted in improved precision and allowed us to draw some slightly different conclusions about the assay combinations. Previous accuracy estimates (see above) indicated that using CRIB146 alone was sufficient to obtain a detection accuracy of 100% (Table 3). New estimates obtained from the simulations showed that this assay produced a lower detection accuracy of 99.66% (resulting from a FP rate of 0.69% i.e. one false positive every 145 samples tested). Combining the two additional *C*. *ribicola*-specific assays decreased the FP rate to one false positive every 714 samples tested and raised the accuracy to 99.93% (Fig 3). Similarly, we also improved precision for the assays targeting *M*. *larici-populina*. The combination of the two available assays was required to reach a maximum accuracy of 99.92% for this species (Table 3). However, from a practical point of view, such a slight increase in accuracy likely would not justify the time and resources required to run additional assays.

## Conclusions

The advantage of using the GEDI approach is that unique genome regions are more likely to provide highly specific assays that can discriminate closely related taxa and are amenable to multiplexing. The disadvantage is that currently the number of sequenced genomes available is still far smaller than the number of DNA sequences available in public databases for the well conserved genes that are used for DNA barcoding and DNA detection. We do foresee, however, a dramatic increase in the number of pathogen genomes that will be available in the future, making the GEDI approach extremely relevant. In addition, future uses of this approach could combine the front portion of the GEDI method with target-enrichment of the target genome regions, followed by high-throughput sequencing, instead of real-time PCR. This approach, already used for human pathogens in clinical settings [52], will likely become the standard in the future for biosurveillance of plant pathogens.

As the proposed assays target some of the most important tree rust pathogens, the adoption of these valuable tools for the monitoring of these fungi should contribute to forest health protection. On a broader perspective, the methodology used in this study provides a framework for the development and validation of robust molecular detection assays that should be widely applicable across different species.

## Supporting information

S1 Fig Venn diagrams showing putative species-specific (A) and genus-specific (B) protein models, as identified by a clustering process with the orthoMCL algorithm. (TIFF)

S1 Table Isolates used for the development of the *Cronartium* genus- and *Cronartium ribicola*-specific assays. (DOCX)

S2 Table Isolates used for the development of the *Melampsora* genus-, *Melampsora medusae* f. sp. *deltoidae*- and *Melampsora larici-populina*-specific assays. (DOCX)

S3 Table Positive detections (C_t_ values < 40.0) for *Cronartium* spp. and *Cronartium ribicola* on environmental samples. (DOCX)

S4 Table Positive detections (C_t_ values < 40.0) for *Melampsora* spp., *Melampsora medusae* f. sp. *deltoidae* and *Melampsora larici-populina* on environmental samples. (DOCX)

S5 Table Species samples used for testing/interpretation of the assays. Values indicate the number of positive (+) and negative (-) samples per species that were tested for each targeted taxonomic group.
(DOCX)

S6 Table Detection limit of the *Cronartium* genus- and *Cronartium ribicola*-specific assays, using a known amount of *Cronartium ribicola* aeciospores from which DNA was extracted. The replicate numbers in the first column correspond to the three independent extractions from the same number of spores.
(DOCX)

S7 Table Detection limit of *Melampsora* genus- and *Melampsora medusae*-specific assays, using a known amount of *Melampsora medusae* f. sp. *deltoidae* urediospores from which DNA was extracted. The replicate numbers in the first column correspond to the three independent extractions from the same number of spores.
(DOCX)

S8 Table Detection limit of *Melampsora* genus- and *Melampsora larici-populina*-specific assays, using a known amount of *Melampsora larici-populina* urediospores from which DNA was extracted. The replicate numbers in the first column correspond to the three independent extractions from the same number of spores.
(DOCX)
