# Supplementary figures and images for: Genome-enhanced detection and identification of fungal pathogens responsible for pine and poplar rust diseases
Source: PLoS One. 2019 Feb 6;14(2):e0210952. doi: 10.1371/journal.pone.0210952 (PMC6364900; doi:10.1371/journal.pone.0210952)

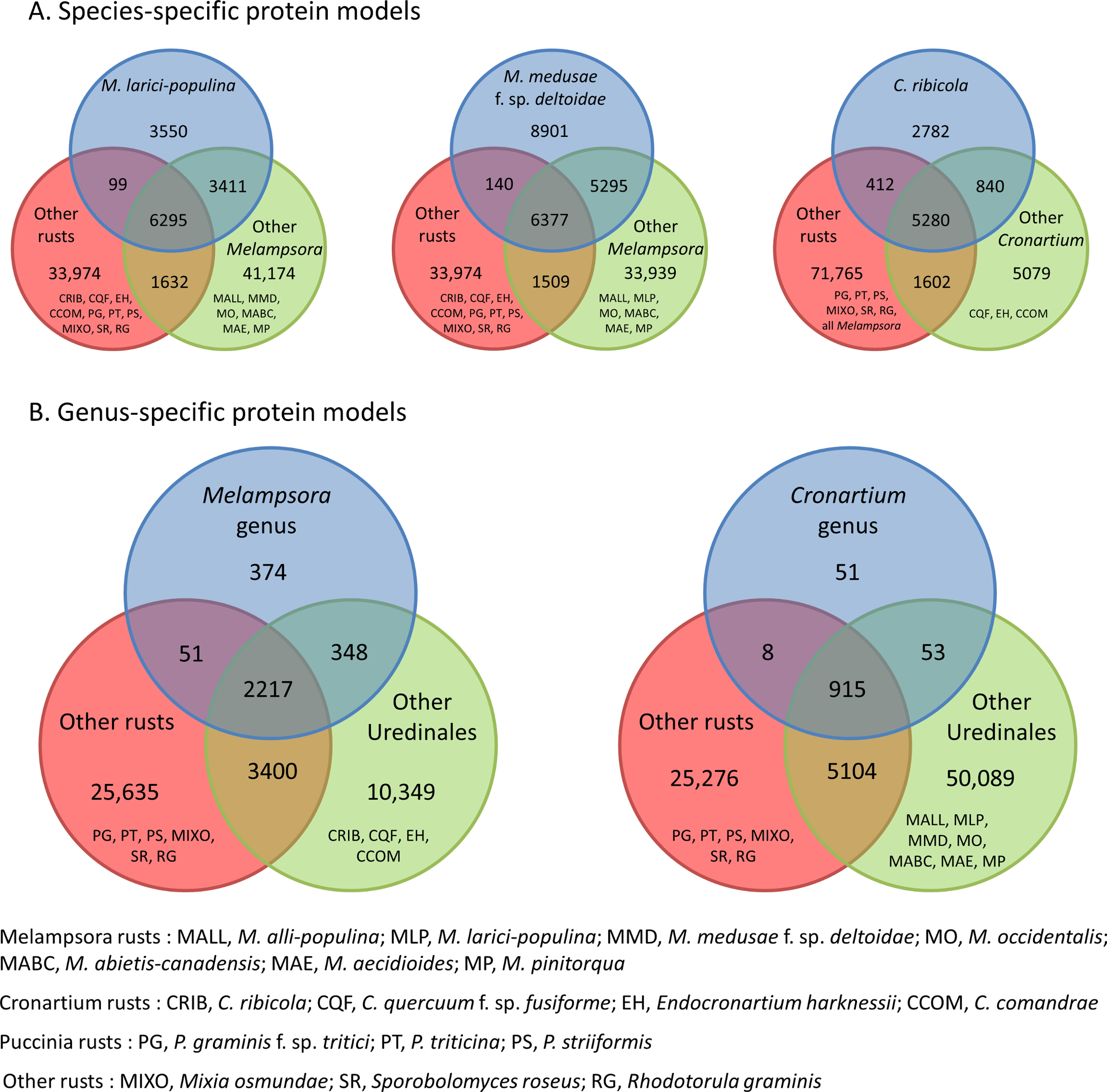

Supplement: S1 Fig — (TIFF) [file pone.0210952.s001.tiff]
