# Supplementary material for: Genome-enhanced detection and identification of fungal pathogens responsible for pine and poplar rust diseases
Source: PLoS One. 2019 Feb 6;14(2):e0210952. doi: 10.1371/journal.pone.0210952 (PMC6364900; doi:10.1371/journal.pone.0210952)
Supplement: S1 Table — (DOCX) [file pone.0210952.s002.docx]

**S1 Table. Isolates used for the development of the *Cronartium* genus- and *Cronartium ribicola*-specific assays.**

|  |  |  |  |  | Geographic coordinates | | Date of | CRO | | CRIB | | |
| --- | --- | --- | --- | --- | --- | --- | --- | --- | --- | --- | --- | --- |
| Species | Isolate (herbarium ID) | ITS or 28S | Sampled material | Provenance | Latitude | Longitude | sampling | 30 | 46 | 65 | 146 | 190 |
| *Cronartium ribicola* | 307^a^ | [CRITS200-18](http://www.barcodinglife.org/index.php/MAS_DataRetrieval_OpenSequence?selectedrecordid=9462380) | Axenic culture of haploid mycelium | CA, USA | 38.739 | -120.742 | n/a | 19.79 | 20.02 | 25.45 | 24.97 | 28.81^c^ |
|  | 20-1^a, b^ | [CRITS201-18](http://www.barcodinglife.org/index.php/MAS_DataRetrieval_OpenSequence?selectedrecordid=9462381) | Axenic culture of haploid mycelium | CA, USA | 38.739 | -120.742 | n/a | 21.98 | 24.19 | 25.44^c^ | 24.99^c^ | 28.83^c^ |
|  | Cr11CAPR30-7^a, b^ | [CRITS202-18](http://www.barcodinglife.org/index.php/MAS_DataRetrieval_OpenSequence?selectedrecordid=9462382) | Uredinial culture of mono-uredinial origin | Cap Rouge, QC, Canada | 46.761 | -71.348 | 2011 | 25.92 | 24.16 | 25.28^c^ | 24.69^c^ | 28.45^c^ |
|  | Cr11Sib15-1^a, b^ |  | Uredinial culture of mono-uredinial origin | Altai Republic, Siberia, Russia | 51.044 | 85.605 | 2011 | 26.39^c^ | 24.93^c^ | 25.37^c^ | 24.84^c^ | 29.00^c^ |
|  | Cr11Sib03-1 | [CRITS203-18](http://www.barcodinglife.org/index.php/MAS_DataRetrieval_OpenSequence?selectedrecordid=9462383) | Uredinial culture of mono-uredinial origin | Tomsk Oblast, Siberia, Russia | 56.310 | 85.174 | 2011 | n/a | n/a | 31.10 | 30.62 | 36.91 |
|  | Cr11Sib20-1 | [CRITS204-18](http://www.barcodinglife.org/index.php/MAS_DataRetrieval_OpenSequence?selectedrecordid=9462384) | Uredinial culture of mono-uredinial origin | Altai Republic, Siberia, Russia | 51.799 | 87.274 | 2011 | n/a | n/a | 29.88 | 29.03 | 33.34 |
|  | Cr11Sib25-1 | [CRITS205-18](http://www.barcodinglife.org/index.php/MAS_DataRetrieval_OpenSequence?selectedrecordid=9462385) | Uredinial culture of mono-uredinial origin | Altai Republic, Siberia, Russia | 51.778 | 87.606 | 2011 | n/a | n/a | 27.38 | 26.86 | 30.90 |
|  | Cr12SS2^a^ | [CRITS206-18](http://www.barcodinglife.org/index.php/MAS_DataRetrieval_OpenSequence?selectedrecordid=9462386) | Uredinial culture of mono-aecial origin | Sainte-Sophie, QC, Canada | 45.866 | -73.881 | 2012 | n/a | n/a | 24.11 | 24.06 | 27.37^c^ |
|  | ID1-4B^a^ |  | Uredinial culture of mono-aecial origin | ID, USA | n/a | n/a | 1999 | n/a | n/a | 26.94^c^ | 26.54^c^ | 30.11^c^ |
|  | WI2-1B^a^ |  | Uredinial culture of mono-aecial origin | WI, USA | n/a | n/a | n/a | n/a | n/a | 25.44^c^ | 25.17^c^ | 28.29^c^ |
|  | WY3-1B^a^ | [CRITS207-18](http://www.barcodinglife.org/index.php/MAS_DataRetrieval_OpenSequence?selectedrecordid=9462387) | Uredinial culture of mono-aecial origin | WY, USA | n/a | n/a | n/a | n/a | n/a | 26.11^c^ | 25.75^c^ | 29.07^c^ |
|  | WI6-1B | [CRITS208-18](http://www.barcodinglife.org/index.php/MAS_DataRetrieval_OpenSequence?selectedrecordid=9462388) | Uredinial culture of mono-aecial origin | WI, USA | n/a | n/a | n/a | n/a | n/a | 22.87 | 22.21 | 25.66 |
|  | VT1-1A | [CRITS088-09](http://www.barcodinglife.org/index.php/MAS_DataRetrieval_OpenSequence?selectedrecordid=1100857) | Uredinial culture of mono-aecial origin | VT, USA | n/a | n/a | 1998 | n/a | n/a | 22.67 | 22.19 | 25.39 |
|  | ME2-9B | [CRITS209-18](http://www.barcodinglife.org/index.php/MAS_DataRetrieval_OpenSequence?selectedrecordid=9462389) | Uredinial culture of mono-aecial origin | ME, USA | n/a | n/a | n/a | n/a | n/a | 24.44 | 24.09 | 27.35 |
|  | OR2-3B | [CRITS210-18](http://www.barcodinglife.org/index.php/MAS_DataRetrieval_OpenSequence?selectedrecordid=9462390) | Uredinial culture of mono-uredinial origin | OR, USA | n/a | n/a | n/a | n/a | n/a | 24.70 | 23.99 | 27.41 |
|  | SD1-3A | [CRITS211-18](http://www.barcodinglife.org/index.php/MAS_DataRetrieval_OpenSequence?selectedrecordid=9462391) | Uredinial culture of mono-aecial origin | SD, USA | n/a | n/a | 1998 | n/a | n/a | 23.95 | 23.40 | 26.81 |
|  | NM42 | [CRITS212-18](http://www.barcodinglife.org/index.php/MAS_DataRetrieval_OpenSequence?selectedrecordid=9462392) | *Pinus strobiformis* | Lincoln National Forest, NM | 32.885 | -105.691 | 1998 | n/a | n/a | 26.93 | 26.43 | 30.17 |
|  | MP-1A | [CRITS213-18](http://www.barcodinglife.org/index.php/MAS_DataRetrieval_OpenSequence?selectedrecordid=9462393) | Aecial blister on *Pinus aristata* | Mosca Pass, CO, USA | 37.629 | -105.550 | 2004 | n/a | n/a | 30.87 | 29.66 | 35.73 |
|  | NY4-1a | [CRITS214-18](http://www.barcodinglife.org/index.php/MAS_DataRetrieval_OpenSequence?selectedrecordid=9462394) | Uredinial culture of mono-uredinial origin | NY, USA | n/a | n/a | 1998 | n/a | n/a | 24.22 | 23.78 | 27.21 |
|  | NH1-1b | [CRITS215-18](http://www.barcodinglife.org/index.php/MAS_DataRetrieval_OpenSequence?selectedrecordid=9462395) | Uredinial culture of mono-aecial origin | NH, USA | n/a | n/a | 1999 | n/a | n/a | 24.34 | 23.98 | 27.50 |
|  | NM1-1b | [CRITS216-18](http://www.barcodinglife.org/index.php/MAS_DataRetrieval_OpenSequence?selectedrecordid=9462396) | Uredinial culture of mono-aecial origin | NM, USA | n/a | n/a | 1998 | n/a | n/a | 24.44 | 23.95 | 27.30 |
|  | GR10-1 | [CRITS217-18](http://www.barcodinglife.org/index.php/MAS_DataRetrieval_OpenSequence?selectedrecordid=9462397) | Unruptured aecial blister on *Pinus strobus* | Gander River, NFLD, Canada | 49.026 | -54.813 | 1995 | n/a | n/a | 29.05 | 28.59 | 33.28 |
|  | TRAF5a | [CRITS218-18](http://www.barcodinglife.org/index.php/MAS_DataRetrieval_OpenSequence?selectedrecordid=9462398) | Unruptured aecial blister on *Pinus strobus* | Trafalgar, NS, Canada | 45.288 | -62.660 | 1995 | n/a | n/a | 29.50 | 29.17 | 33.98 |
|  | NB11-B | [CRITS219-18](http://www.barcodinglife.org/index.php/MAS_DataRetrieval_OpenSequence?selectedrecordid=9462399) | Unruptured aecial blister on *Pinus strobus* | Moncton, NB, Canada | 46.116 | -64.803 | 1995 | n/a | n/a | 27.10 | 26.59 | 30.94 |
|  | MI-2A | [CRITS220-18](http://www.barcodinglife.org/index.php/MAS_DataRetrieval_OpenSequence?selectedrecordid=9462400) | Unruptured aecial blister on *Pinus strobus* | Minden, ON, Canada | 44.925 | -78.724 | 1995 | n/a | n/a | 30.15 | 29.62 | 33.82 |
|  | S-3A | [CRITS221-18](http://www.barcodinglife.org/index.php/MAS_DataRetrieval_OpenSequence?selectedrecordid=9462401) | Unruptured aecial blister on *Pinus strobus* | Sault-Sainte-Marie, ON, Canada | 46.533 | -84.350 | 1995 | n/a | n/a | 31.32 | 30.77 | 36.06 |
|  | 95SCB-2C | [CRITS222-18](http://www.barcodinglife.org/index.php/MAS_DataRetrieval_OpenSequence?selectedrecordid=9462402) | Unruptured aecial blister on *Pinus strobus* | Ste-Camille-de-Bellechasse, QC | 46.494 | -70.219 | 1995 | n/a | n/a | 28.39 | 28.18 | 31.86 |
|  | 96CO2D | [CRITS223-18](http://www.barcodinglife.org/index.php/MAS_DataRetrieval_OpenSequence?selectedrecordid=9462403) | Unruptured aecial blister on *Pinus strobus* | Corte-Réal, QC, Canada | 48.908 | -64.600 | 1996 | n/a | n/a | 30.11 | 29.93 | 34.18 |
|  | MC-6C |  | Unruptured aecial blister on *Pinus strobus* | Lac aux Araignées, QC | 45.450 | -70.769 | 1993 | n/a | n/a | 27.41 | 27.01 | 31.49 |
|  | Q1-1 |  | Unruptured aecial blister | Quartz Gravel Pit, Golden, BC | 51.490 | -117.368 | n/a | n/a | n/a | 34.86 | 33.53 | 39.61 |
|  | Cr France^a^ | [CRITS072-09](http://www.barcodinglife.org/index.php/MAS_DataRetrieval_OpenSequence?selectedrecordid=1100841) | *Ribes* sp. | France | n/a | n/a | n/a | n/a | n/a | 29.49^c^ | 29.00^c^ | 33.20^c^ |
|  | CR3 | [CRITS071-09](http://www.barcodinglife.org/index.php/MAS_DataRetrieval_OpenSequence?selectedrecordid=1100840) | Opened aecial blister on *Pinus strobus* | Finland, Helsinki | 60.220 | 25.002 | 2003 | n/a | n/a | 26.01 | 25.37 | 29.38 |
|  | P14^a^ | [CRITS079-09](http://www.barcodinglife.org/index.php/MAS_DataRetrieval_OpenSequence?selectedrecordid=1100848) | *Pinus koraiensis* | Pyongchang county, Korea | 37.501 | 128.509 | 2004 | 28.21 | 27.32 | 25.95^c^ | 25.74^c^ | UNDETM |
|  | P4 | [CRITS074-09](http://www.barcodinglife.org/index.php/MAS_DataRetrieval_OpenSequence?selectedrecordid=1100843) | *Pinus koraiensis* | Pyongchang county, Korea | 37.501 | 128.509 | 2004 | n/a | n/a | 30.67 | 31.00 | UNDETM |
|  | P5-2 | [CRITS075-09](http://www.barcodinglife.org/index.php/MAS_DataRetrieval_OpenSequence?selectedrecordid=1100844) | *Pinus koraiensis* | Pyongchang county, Korea | 37.501 | 128.509 | 2004 | 30.67 | 29.35 | 29.44 | 29.45 | UNDETM |
|  | P6-3 | [CRITS076-09](http://www.barcodinglife.org/index.php/MAS_DataRetrieval_OpenSequence?selectedrecordid=1100845) | *Pinus koraiensis* | Pyongchang county, Korea | 37.501 | 128.509 | 2004 | n/a | n/a | 32.13 | 31.98 | UNDETM |
|  | Y2-2 | [CRITS082-09](http://www.barcodinglife.org/index.php/MAS_DataRetrieval_OpenSequence?selectedrecordid=1100851) | *Pinus koraiensis* | Yangpyong county, Korea | 38.176 | 128.104 | 2004 | 29.13 | 28.25 | 28.07 | 28.14 | UNDETM |
| *Cronartium comandrae* | C2^a, b^ | [CRITS115-09](http://www.barcodinglife.org/index.php/MAS_DataRetrieval_OpenSequence?selectedrecordid=1100884) | Axenic culture of mycelium | ND, USA | n/a | n/a | n/a | 24.28^c^ | 23.60 | UNDETM | UNDETM | UNDETM |
|  | LP 3A^a, b^ |  | *Pinus banksiana* | Lac Saint-Jean, QC, Canada | 49.138 | -73.409 | 2001 | 28.00 | 24.63 | UNDETM | UNDETM | UNDETM |
|  | MG-3^a, b^ | [CRITS132-09](http://www.barcodinglife.org/index.php/MAS_DataRetrieval_OpenSequence?selectedrecordid=1100901) | n/a | Mugaha Marsh, BC, Canada | 55.387 | -123.173 | 2006 | 29.36 | 25.34 | UNDETM | UNDETM | UNDETM |
| *Cronartium quercuum* f. sp. *fusiforme* | CqfF2^a, b^ | [CRITS144-09](http://www.barcodinglife.org/index.php/MAS_DataRetrieval_OpenSequence?selectedrecordid=1100913) | *Pinus taeda* | USA | n/a | n/a | 1991 | 30.14^c^ | 24.11^c^ | UNDETM | UNDETM | UNDETM |
|  | CqfH2^a, b^ | [CRITS146-09](http://www.barcodinglife.org/index.php/MAS_DataRetrieval_OpenSequence?selectedrecordid=1100915) | *Pinus taeda* | SC, USA | n/a | n/a | 1991 | 25.94^c^ | 24.86 | UNDETM | UNDETM | UNDETM |
|  | CqfR4^a, b^ | [CRITS149-09](http://www.barcodinglife.org/index.php/MAS_DataRetrieval_OpenSequence?selectedrecordid=1100918) | *Pinus taeda* | TX, USA | n/a | n/a | 1991 | 26.37^c^ | 25.30^c^ | UNDETM | UNDETM | UNDETM |
| *Endocronartium harknessii* | PhW7WM^a, b^ | [CRITS224-18](http://www.barcodinglife.org/index.php/MAS_DataRetrieval_OpenSequence?selectedrecordid=9462404) | Axenic culture of mycelium | McHenry County, ND, USA | 48.343 | -100.701 | n/a | 29.48 | 25.37 | UNDETM | UNDETM | UNDETM |
|  | Alas1^a, b^ | [CRITS169-09](http://www.barcodinglife.org/index.php/MAS_DataRetrieval_OpenSequence?selectedrecordid=1100938) | *Pinus* sp. | AK, USA | n/a | n/a | 2006 | 27.83 | 24.11 | UNDETM | UNDETM | UNDETM |
|  | SU T2-1^a, b^ | [CRITS187-09](http://www.barcodinglife.org/index.php/MAS_DataRetrieval_OpenSequence?selectedrecordid=1100956) | *Pinus banksiana* | Saint-Urbain, QC, Canada | 47.557 | -70.539 | 2003 | 27.43^c^ | 26.54^c^ | UNDETM | UNDETM | UNDETM |
| *Cronartium quercuum* f. sp. *banksianae* | CqE3^a, b^ | [CRITS196-09](http://www.barcodinglife.org/index.php/MAS_DataRetrieval_OpenSequence?selectedrecordid=1100965) | Axenic culture of mycelium | Hubbard County, MN, USA | 47.177 | -94.943 | n/a | 28.54^c^ | 27.03^c^ | UNDETM | UNDETM | UNDETM |
|  | CqE7^a, b^ | [CRITS198-09](http://www.barcodinglife.org/index.php/MAS_DataRetrieval_OpenSequence?selectedrecordid=1100967) | Axenic culture of mycelium | Hubbard County, MN, USA | 47.177 | -94.943 | n/a | 23.91^c^ | 23.55 | UNDETM | UNDETM | UNDETM |
|  | CqE9^a, b^ | [CRITS199-09](http://www.barcodinglife.org/index.php/MAS_DataRetrieval_OpenSequence?selectedrecordid=1100968) | Axenic culture of mycelium | Hubbard County, MN, USA | 47.177 | -94.943 | n/a | 25.86^c^ | 24.30 | UNDETM | UNDETM | UNDETM |
| *Cronartium* sp. on *Pinus armandii* | Sic1-3-1a | [CRITS095-09](http://www.barcodinglife.org/index.php/MAS_DataRetrieval_OpenSequence?selectedrecordid=1100864) | *Pinus armandii* | Sichuan province, China | 30.103 | 102.800 | n/a | 30.25^c^ | 29.10^c^ | 32.60 | UNDETM | UNDETM |
|  | Sic1-8-1c | [CRITS103-09](http://www.barcodinglife.org/index.php/MAS_DataRetrieval_OpenSequence?selectedrecordid=1100872) | *Pinus armandii* | Sichuan province, China | 30.103 | 102.800 | n/a | 33.58 | 31.51 | 34.45 | UNDETM | UNDETM |
|  | Sic1-9-1a | [CRITS104-09](http://www.barcodinglife.org/index.php/MAS_DataRetrieval_OpenSequence?selectedrecordid=1100873) | *Pinus armandii* | Sichuan province, China | 30.103 | 102.800 | n/a | 28.97^c^ | 28.55^c^ | 31.20 | UNDETM | UNDETM |
| *Cronartium comptoniae* | 167 7A^a, b^ | [CRITS159-09](http://www.barcodinglife.org/index.php/MAS_DataRetrieval_OpenSequence?selectedrecordid=1100928) | *Pinus banksiana* | Lac Saint-Jean, QC, Canada | 48.792 | -72.722 | 2001 | 28.60^c^ | 27.24 | UNDETM | UNDETM | UNDETM |
| *Cronartium quercuum* f. sp*. virginianae* | CqCNincB1^a, b^ | [CRITS152-09](http://www.barcodinglife.org/index.php/MAS_DataRetrieval_OpenSequence?selectedrecordid=1100921) | *Quercus velutina* | NC, USA | n/a | n/a | n/a | 28.45^c^ | 27.40 | UNDETM | UNDETM | UNDETM |
| *Cronartium flaccidum* | 1112 (QFB 25391)^a, b^ | [CRITS047-09](http://www.barcodinglife.org/index.php/MAS_DataRetrieval_OpenSequence?selectedrecordid=1065559) | *Melampyrum* sp. | Cornebarrieu, France | 43.646 | 1.260 | 2008 | 25.95^c^ | 25.12 | UNDETM | UNDETM | UNDETM |
| *Endocronartium pini* | Jokkmokk-3^a, b^ |  | *Pinus* sp. | Jokkmokk, Sweden | 66.605 | 19.823 | 2012 | 28.65^c^ | 27.98 | UNDETM | UNDETM | UNDETM |
| *Cronartium strobilinum* | G317 HGS1^a, b^ | [CRITS139-09](http://www.barcodinglife.org/index.php/MAS_DataRetrieval_OpenSequence?selectedrecordid=1100908) | *Pinus taeda* | FL, USA | 29.633 | -82.372 | 2004 | 28.43 | 27.62 | UNDETM | UNDETM | UNDETM |
| *Cronartium coleosporioides* | Ccol yh2^a, b^ | [CRITS155-09](http://www.barcodinglife.org/index.php/MAS_DataRetrieval_OpenSequence?selectedrecordid=1100924) | n/a | AB, Canada | n/a | n/a | n/a | 24.57 | 24.00 | UNDETM | UNDETM | UNDETM |
| *Melampsora medusae* f. sp. *deltoidae* | 761 (QFB 25044)^a, b^ | [MPITS053-08](http://www.barcodinglife.org/index.php/MAS_DataRetrieval_OpenSequence?selectedrecordid=932335) | *Populus deltoides* | Saints-Anges, QC, Canada | 46.416 | -70.876 | 2007 | UNDETM | UNDETM | UNDETM | UNDETM | UNDETM |
|  | 98-SU-10-2a |  | Single uredium on *Populus* sp. ?? | Sutton, QC, Canada | 45.105 | -72.616 | 1998 | UNDETM | UNDETM | UNDETM | UNDETM | UNDETM |
|  | 98-OT-4-1b |  | Single uredium on *Populus* sp. ?? | Ottawa, ON, Canada | 45.420 | -75.697 | 1998 | UNDETM | UNDETM | n/a | n/a | n/a |
|  | 98-MS-5-3a |  | Single uredium on *Populus* sp. ?? | MS, USA | n/a | n/a | 1998 | UNDETM | UNDETM | n/a | n/a | n/a |
|  | 04-SF-Ec-20 |  | Single aecium | Saint-Félicien, QC, Canada | 48.650 | -72.449 | 2004 | UNDETM | UNDETM | n/a | n/a | n/a |
| *Melampsora* larici-populina | 297 (QFB 25099)^a, b^ | [MPITS009-08](http://www.barcodinglife.org/index.php/MAS_DataRetrieval_OpenSequence?selectedrecordid=932291) | *Populus* sp. | Lotbinière, QC, Canada | 46.488 | -71.926 | 2006 | UNDETM | UNDETM | UNDETM | UNDETM | UNDETM |
|  | 880 (MRNFQ 32347) | [MPITS075-08](http://www.barcodinglife.org/index.php/MAS_DataRetrieval_OpenSequence?selectedrecordid=932357) | *Larix decidua* | Saint-Modeste, QC, Canada | 47.839 | -69.392 | 2007 | UNDETM | UNDETM | UNDETM | UNDETM | UNDETM |
|  | 747210B5-1 |  | *Populus* sp. | Normandin, QC, Canada | 48.767 | -72.533 | 2004 | UNDETM | UNDETM | UNDETM | UNDETM | UNDETM |
| *Melampsora occidentalis* | 376 (QFB 25117)^b^ | [MPITS016-08](http://www.barcodinglife.org/index.php/MAS_DataRetrieval_OpenSequence?selectedrecordid=932298) | *Populus* sp. | Vancouver, BC, Canada | 49.249 | -123.228 | 2006 | UNDETM | UNDETM | n/a | n/a | n/a |
|  | Mo05CA07 | [MPITS191-18](http://www.barcodinglife.org/index.php/MAS_DataRetrieval_OpenSequence?selectedrecordid=9494379) | n/a | CA, USA | n/a | n/a | 2005 | UNDETM | UNDETM | n/a | n/a | n/a |
| *Melampsora abietis-canadensis* | 666 (QFB 25029)^b^ | [MACOX008-08](http://www.barcodinglife.org/index.php/MAS_DataRetrieval_OpenSequence?selectedrecordid=761397) | *Tsuga canadensis* | Saint-Hippolyte, QC, Canada | 45.957 | -74.001 | 2007 | UNDETM | UNDETM | n/a | n/a | n/a |
|  | 1400 (PUR 61512) | [MPITS161-09](http://www.barcodinglife.org/index.php/MAS_DataRetrieval_OpenSequence?selectedrecordid=1082952) | *Populus grandidentata* | WI, USA | n/a | n/a | 1959 | UNDETM | UNDETM | UNDETM | UNDETM | UNDETM |
|  | MEA CAP1-1AT7 | [PRJNA365810](https://www.ncbi.nlm.nih.gov/bioproject/365810) |  | Cap Tourmente, QC, Canada | 47.081 | -70.778 | 2009 | UNDETM | UNDETM | n/a | n/a | n/a |
| *Melampsora allii-populina* | 1260 (DAOM 216857)^b^ | [MPITS112-08](http://www.barcodinglife.org/index.php/MAS_DataRetrieval_OpenSequence?selectedrecordid=932394) | *Populus canadensis* | Hungary | 47.330 | 19.870 | 1988 | UNDETM | UNDETM | n/a | n/a | n/a |
|  | PFH03-23 (QFB 25064) | [JN881731](https://www.ncbi.nlm.nih.gov/nuccore/jN881731) | *Populus nigra* | Pazardjik, Bulgaria | 42.183 | 24.330 | 2003 | UNDETM | UNDETM | UNDETM | UNDETM | UNDETM |
|  | PFH04-9 (QFB 25513) | [JN881729](https://www.ncbi.nlm.nih.gov/nuccore/JN881729) | *Populus nigra* | Madrid, Spain | 40.452 | -3.741 | 2004 | UNDETM | UNDETM | UNDETM | UNDETM | UNDETM |
| *Melampsora aecidioides* | 380 (QFB 25017)^b^ | [EU808041](https://www.ncbi.nlm.nih.gov/nuccore/EU808041.1) | *Populus alba* | Vancouver, BC, Canada | 49.274 | -123.228 | 2006 | UNDETM | UNDETM | n/a | n/a | n/a |
|  | 664 (QFB 25028) | [MAITS005-08](http://www.barcodinglife.org/index.php/MAS_DataRetrieval_OpenSequence?selectedrecordid=761475) | *Populus alba* | Colquitz creek, BC, Canada | 48.459 | -123.394 | 2007 | UNDETM | UNDETM | UNDETM | UNDETM | UNDETM |
| *Melampsora pinitorqua* | Mpini7^b^ | [PRJNA190833](https://www.ncbi.nlm.nih.gov/bioproject/PRJNA190833) | n/a | France | n/a | n/a | n/a | UNDETM | UNDETM | n/a | n/a | n/a |
| *Melampsora epitea* | 685A^b^ | [JF825969](https://www.ncbi.nlm.nih.gov/nuccore/jf825969) | *Salix viminalis* | Svalöv, Sweden | 55.913 | 13.102 | 1996 | UNDETM | UNDETM | n/a | n/a | n/a |
| *Coleosporium viburni* | 160 | [COITS001-08](http://www.barcodinglife.org/index.php/MAS_DataRetrieval_OpenSequence?selectedrecordid=932230) | *Pinus resinosa* | Estrie, QC, Canada | 45.424 | -71.223 | 1998 | UNDETM | UNDETM | UNDETM | UNDETM | UNDETM |
| *Coleosporium asterum* | 237 (QFB 25087) | [COITS033-18](http://www.barcodinglife.org/index.php/MAS_DataRetrieval_OpenSequence?selectedrecordid=9502759) | *Solidago* sp. | Le Bic, QC, Canada | 48.375 | -68.695 | 2006 | UNDETM | UNDETM | UNDETM | UNDETM | UNDETM |
| *Coleosporium tussilaginis* | 1097 (QFB 25340) |  | *Senecio vulgaris* | Cornebarrieu, France | 42.652 | 1.314 | 2008 | UNDETM | UNDETM | UNDETM | UNDETM | UNDETM |
| *Coleosporium pinicola* | 550 (DAOM 213222) |  | *Pinus contorta* | Bonaventure, QC, Canada | 48.049 | -65.484 | 1991 | UNDETM | UNDETM | n/a | n/a | n/a |
| *Pucciniastrum vaccinii* | 231 (H1249) |  | *Tsuga* sp. | Sainte-Jeanne-d'Arc, QC | 45.712 | -72.475 | 1986 | UNDETM | UNDETM | UNDETM | UNDETM | UNDETM |
|  | 531 (DAOM 97346) | [PMITS041-08](http://www.barcodinglife.org/index.php/MAS_DataRetrieval_OpenSequence?selectedrecordid=934613) | *Vaccinium deliciosum* | Burman Lake, BC, Canada | 49.642 | -125.745 | 1961 | UNDETM | UNDETM | UNDETM | UNDETM | UNDETM |
| *Pucciniastrum americanum* | 260 barcode | [PMITS045-18](http://www.barcodinglife.org/index.php/MAS_DataRetrieval_OpenSequence?selectedrecordid=9478037) | n/a | Portneuf, QC, Canada | 46.691 | -71.888 | 1998 | 41.61 | 36.7^d^ | UNDETM | UNDETM | UNDETM |
| *Pucciniastrum agrimoniae* | 369 barcode | [PMITS013-08](http://www.barcodinglife.org/index.php/MAS_DataRetrieval_OpenSequence?selectedrecordid=934585) | *Agrimonia gryposepala* | Québec, QC, Canada | 46.798 | -71.221 | 2006 | 32.83^d^ | UNDETM | UNDETM | UNDETM | UNDETM |
| *Pucciniastrum goeppertianum* | 232 barcode |  | *Abies* sp. | Valcartier, QC, Canada | 46.947 | -71.495 | 2006 | UNDETM | UNDETM | n/a | n/a | n/a |
| *Pucciniastrum epilobii* | 265 barcode | [PMITS016-08](http://www.barcodinglife.org/index.php/MAS_DataRetrieval_OpenSequence?selectedrecordid=934588) | *Epilobium* sp. | Zec des Martres, QC, Canada | 47.753 | -70.656 | 2006 | UNDETM | UNDETM | n/a | n/a | n/a |
| *Chrysomyxa ledicola* | 636 (QFB 25135) | [CHITS121-18](http://www.barcodinglife.org/index.php/MAS_DataRetrieval_OpenSequence?selectedrecordid=9462115) | *Rhododendron groenlandicum* | Zec des Martres, QC, Canada | 47.753 | -70.656 | 2007 | UNDETM | UNDETM | UNDETM | UNDETM | UNDETM |
| *Chrysomyxa pirolata* | 920 (QFB 25056) | [CHITS066-08](http://www.barcodinglife.org/index.php/MAS_DataRetrieval_OpenSequence?selectedrecordid=906818) | *Pyrola* sp. | Bic, QC, Canada | 48.359 | -68.768 | 2008 | UNDETM | UNDETM | UNDETM | UNDETM | UNDETM |
| *Chrysomyxa cassandrae* | 199 (QFB 25005) | [CHITS052-08](http://www.barcodinglife.org/index.php/MAS_DataRetrieval_OpenSequence?selectedrecordid=905474) | *Picea mariana* | Abitibi, QC, Canada | 47.951 | -77.997 | 2004 | UNDETM | UNDETM | n/a | n/a | n/a |
| *Chrysomyxa chiogenis* | 224 (QFB 25008) | [CHITS005-08](http://www.barcodinglife.org/index.php/MAS_DataRetrieval_OpenSequence?selectedrecordid=766310) | *Gaulteria hispidula* | Zec des Martres, QC, Canada | 47.753 | -70.657 | 2006 | UNDETM | UNDETM | n/a | n/a | n/a |
| *Chrysomyxa weirii* | 545 (QFB 25018) | [CHITS014-08](http://www.barcodinglife.org/index.php/MAS_DataRetrieval_OpenSequence?selectedrecordid=766319) | *Picea* sp. | Guelph, ON, Canada | 43.531 | -80.226 | 2007 | UNDETM | UNDETM | n/a | n/a | n/a |
|  | 916 (QFB 25269) | [CHITS080-08](http://www.barcodinglife.org/index.php/MAS_DataRetrieval_OpenSequence?selectedrecordid=906832) | *Picea glauca* | Notre-Dame-des-Pins, QC | 46.181 | -70.711 | 2008 | UNDETM | UNDETM | n/a | n/a | n/a |
| *Chrysomyxa woroninii* | 643 (QFB 25141) | [CHITS086-09](http://www.barcodinglife.org/index.php/MAS_DataRetrieval_OpenSequence?selectedrecordid=1100273) | *Rhododendron groenlandicum* | Zec des Martres, QC, Canada | 47.753 | -70.656 | 2007 | UNDETM | UNDETM | n/a | n/a | n/a |
| *Chrysomyxa empetri* | 755 (QFB 25033) | [CHITS032-08](http://www.barcodinglife.org/index.php/MAS_DataRetrieval_OpenSequence?selectedrecordid=766337) | *Empetrum nigrum* | Radisson, QC, Canada | 53.76 | -77.569 | 2007 | UNDETM | UNDETM | n/a | n/a | n/a |

UNDETM : C_t_ value = Undetermined; n/a : not available or untested.

^a^ DNA samples from target and non-target species among the Pucciniales, forming the reduced panel, used for the selection of primer pairs specific to *C. ribicola*.

^b^ DNA samples from target and non-target species among the Pucciniales, forming the reduced panel, used for the selection of primer pairs specific to the genus *Cronartium*.

^c^ Sequence deposited in GenBank.

^d^ means that one of the two technical replicates = UNDETM.
