# Supplementary material for: Genome-enhanced detection and identification of fungal pathogens responsible for pine and poplar rust diseases
Source: PLoS One. 2019 Feb 6;14(2):e0210952. doi: 10.1371/journal.pone.0210952 (PMC6364900; doi:10.1371/journal.pone.0210952)
Supplement: S2 Table — (DOCX) [file pone.0210952.s003.docx]

**S2 Table. Isolates used for the development of the *Melampsora* genus-, *Melampsora medusae* f. sp. *deltoidae*- and *Melampsora larici-populina*-specific assays.**

|  |  |  |  |  | Geographic coordinates | | Date of | MEL | | | MM | | MLP | |
| --- | --- | --- | --- | --- | --- | --- | --- | --- | --- | --- | --- | --- | --- | --- |
| Species | Isolate | ITS or 28S | Sampled material | Provenance | Latitude | Longitude | sampling | 40 | 100 | 176 | 53 | 74 | 104 | 133 |
| *Melampsora medusae* f. sp. *deltoidae* | CR-7a^a^ |  | Uredinial culture of mono-uredinial origin | Cap Rouge, QC, Canada | 46.765 | -71.355 | 1997 | 24.51 | 23.66 | 23.75 | 23.69 | 24.99 | UNDETM | UNDETM |
|  | 583 barcode^a, c^ | [MPITS025-08](http://www.barcodinglife.org/index.php/MAS_DataRetrieval_OpenSequence?selectedrecordid=932307) | *Larix laricina* | Beauharnois, QC, Canada | 45.118 | -74.293 | 2007 | 33.62^d^ | 33.26^d^ | 32.92^d^ | 31.73^d^ | 33.74^d^ | n/a | n/a |
|  | 760 barcode^a, b, c^ | [MPITS052-08](http://www.barcodinglife.org/index.php/MAS_DataRetrieval_OpenSequence?selectedrecordid=932334) | *Populus deltoides* | Saints-Anges, QC, Canada | 46.416 | -70.876 | 2007 | 31.03^d^ | 30.77^d^ | 29.96^d^ | 28.95^d^ | 31.08^d^ | UNDETM | UNDETM |
|  | 98-OT-4-1b^a, c^ |  | Single uredium on *Populus* sp. ?? | Ottawa, ON, Canada | 45.420 | -75.697 | 1998 | 29.45^d^ | 29.03^d^ | 29.37^d^ | 27.73^d^ | 31.69^d^ | UNDETM | UNDETM |
|  | 98-TR-10-3a^a^ |  | Single uredium on *Populus* sp. ?? | Trois-Rivières, QC, Canada | 46.339 | -72.541 | 1998 | n/a | n/a | n/a | 32.28^d^ | 33.84 | n/a | n/a |
|  | 98-SU-10-2a^a^ |  | Single uredium on *Populus* sp. ?? | Sutton, QC, Canada | 45.105 | -72.616 | 1998 | n/a | n/a | n/a | 31.48^d^ | 35.91^d^ | n/a | n/a |
|  | 98-MS-5-3a^a^ |  | Single uredium on *Populus* sp. ?? | MS, USA | n/a | n/a | 1998 | n/a | n/a | n/a | 28.24^d^ | 29.55^d^ | n/a | n/a |
|  | 98D9^a^ |  | Uredinial culture of mono-uredinial origin | South Africa | n/a | n/a | 1998 | n/a | n/a | n/a | 27.44^d^ | 30.55^d^ | n/a | n/a |
| *Melampsora medusae* f. sp. *tremuloidae* | 1028 (QFB 25066)^a, c^ | [MPITS095-08](http://www.barcodinglife.org/index.php/MAS_DataRetrieval_OpenSequence?selectedrecordid=932377) | *Larix laricina* | Péribonka, QC, Canada | 48.784 | -71.998 | 2008 | 29.84^d^ | 29.21^d^ | 28.62^d^ | 27.52^d^ | 30.27^d^ | UNDETM | UNDETM |
|  | BrancheO-B^a^ |  | Uredinial culture of mono-uredinial origin | La Doré, QC, Canada | 48.688 | -72.713 | 2004 | n/a | n/a | n/a | 34.19^d^ | 33.27^d^ | n/a | n/a |
|  | BeauceSt-Zac-B^a^ |  | Uredinial culture of mono-uredinial origin | Saint-Zacharie, QC, Canada | 46.126 | -70.364 | 2004 | n/a | n/a | n/a | 29.28^d^ | 30.65^d^ | n/a | n/a |
|  | BeauceSt-Zac-C^a, c^ |  | Uredinial culture of mono-uredinial origin | Saint-Zacharie, QC, Canada | 46.126 | -70.364 | 2004 | 32.50^d^ | 33.11^d^ | 32.74^d^ | 35.14 | 34.90 | UNDETM | UNDETM |
|  | PP1B1F1A^a, c^ |  | Single uredium on *Populus tremuloides* | Pointe-Platon, QC, Canada | 46.665 | -71.859 | 2003 | 33.94 | 33.08^d^ | 33.13^d^ | 33.00^d^ | 33.52^d^ | UNDETM | UNDETM |
| *Melampsora* *occidentalis* | Mo05CA07^a, b, c^ | [MPITS191-18](http://www.barcodinglife.org/index.php/MAS_DataRetrieval_OpenSequence?selectedrecordid=9494379) | Single uredium or telium | CA, USA | n/a | n/a | 2005 | 23.85^d^ | 22.93^d^ | 23.04^d^ | UNDETM | UNDETM | UNDETM | UNDETM |
| *Melampsora × columbiana* | Mo96D1^a^ |  | Single uredium or telium | Puyallup, WA, USA | 47.184 | -122.292 | 1996 | 36.70 | 35.35 | 35.85 | 34.47 | UNDETM | n/a | n/a |
|  | Mo96E2^a, c^ |  | Single uredium or telium | Puyallup, WA, USA | 47.184 | -122.292 | 1996 | 34.17 | 33.68 | 33.08 | 35.02 | UNDETM | n/a | n/a |
|  | Mo02A1^a, c^ | [MPITS192-18](http://www.barcodinglife.org/index.php/MAS_DataRetrieval_OpenSequence?selectedrecordid=9494380) | Single uredium or telium | Lapwai Canyon, ID, USA | n/a | n/a | 2002 | 34.49^d^ | 33.32^d^ | 33.39^d^ | 32.42 | UNDETM | UNDETM | UNDETM |
|  | Mxc96B1^a^ |  | Single uredium or telium | Clatskanie, OR, USA | 46.103 | -123.204 | 1996 | 36.18 | 35.74 | n/a | 35.22 | UNDETM | UNDETM | UNDETM |
|  | Mxc96C1 | [MPITS193-18](http://www.barcodinglife.org/index.php/MAS_DataRetrieval_OpenSequence?selectedrecordid=9494381) | Single uredium or telium | Clatskanie, OR, USA | 46.103 | -123.204 | 1996 | 36.72 | 35.34 | 35.05 | 37.72 | UNDETM | n/a | n/a |
|  | Mxc96D1^a^ |  | Single uredium or telium | USA | n/a | n/a | 1996 | 35.33 | 36.20 | 35.00 | 37.11 | UNDETM | n/a | n/a |
| *Melampsora larici-populina* | 880 (MRNFQ 32347)^a, b, c^ | [MPITS075-08](http://www.barcodinglife.org/index.php/MAS_DataRetrieval_OpenSequence?selectedrecordid=932357) | *Larix decidua* | Saint-Modeste, QC, Canada | 47.839 | -69.392 | 2007 | 28.98 | 27.46^d^ | 27.34^d^ | UNDETM | 39.15 | 26.95^d^ | 27.57^d^ |
|  | G-P 915313B18-1^a, c^ |  | Uredinial culture of mono-uredinial origin | Grandes-Piles, QC, Canada | 46.680 | -72.690 | 2004 | 31.70^d^ | 29.92^d^ | 29.49 | UNDETM | UNDETM | 29.24 | 30.92 |
|  | Lotb 3675-05^a, c^ |  | Uredinial culture of mono-uredinial origin | Lotbinière, QC, Canada | 46.488 | -71.926 | 2004 | 31.62^d^ | 29.51^d^ | 29.34^d^ | UNDETM | UNDETM | 28.44 | 29.82 |
|  | NZ-04-HB^b^ |  | Uredinial culture of mono-uredinial origin | Hawke’s Bay, New Zealand | -39.113 | 176.742 | 2004 | n/a | n/a | n/a | n/a | n/a | 34.00^d^ | 34.86^d^ |
|  | 837 (QFB 25043)^b^ | [MPITS067-08](http://www.barcodinglife.org/index.php/MAS_DataRetrieval_OpenSequence?selectedrecordid=932349) | *Populus balsamifera* | Villeroy, QC, Canada | 46.401 | -71.910 | 2007 | n/a | n/a | n/a | n/a | n/a | 34.16 | 34.97 |
|  | 1420 (TSH-R16927)^b^ | [MPITS181-09](http://www.barcodinglife.org/index.php/MAS_DataRetrieval_OpenSequence?selectedrecordid=1082972) | *Populus cathayana* | Gansu province, China | n/a | n/a | 2000 | n/a | n/a | n/a | n/a | n/a | 34.07 | 35.84 |
|  | 747210B5-1^b^ |  | Uredinial culture of mono-uredinial origin | Normandin, QC, Canada | 48.767 | -72.533 | 2004 | n/a | n/a | n/a | n/a | n/a | 26.62^d^ | 27.43^d^ |
|  | 18112^b^ |  | Uredinial culture of mono-uredinial origin | Portage-du-Fort, QC, Canada | 45.593 | -76.660 | 2004 | n/a | n/a | n/a | n/a | n/a | 27.68^d^ | 27.93^d^ |
|  | A2F1^b^ |  | Uredinial culture of mono-uredinial origin | Windsor, QC, Canada | 45.569 | -72.007 | 2004 | n/a | n/a | n/a | n/a | n/a | 27.46^d^ | 28.30^d^ |
|  | 17940a^b^ |  | Uredinial culture of mono-uredinial origin | Lavaltrie, QC, Canada | 45.878 | -73.285 | 2004 | n/a | n/a | n/a | n/a | n/a | 28.32^d^ | 29.36^d^ |
|  | 915313PM-1^b^ |  | Uredinial culture of mono-uredinial origin | Sainte-Geneviève-de-Berthier, QC | 46.093 | -73.226 | 2004 | n/a | n/a | n/a | n/a | n/a | 29.19^d^ | 30.41^d^ |
|  | 18716^b^ |  | Uredinial culture of mono-uredinial origin | Sainte-Luce, QC, Canada | 48.515 | -68.373 | 2004 | n/a | n/a | n/a | n/a | n/a | 29.70^d^ | 30.58^d^ |
|  | 18455^b^ |  | Uredinial culture of mono-uredinial origin | Saint-Modeste, QC, Canada | 47.839 | -69.392 | 2004 | n/a | n/a | n/a | n/a | n/a | 28.04^d^ | 29.47^d^ |
| *Melampsora larici-tremulae* | 1461 barcode^a, c^ | [JQ664559](https://www.ncbi.nlm.nih.gov/nuccore/JQ664559) | *Populus tremula* | Meurthe-et-Moselle, France | 48.751 | 6.341 | 1986 | 29.09^d^ | 28.82^d^ | 28.23^d^ | UNDETM | UNDETM | UNDETM | UNDETM |
| *Melampsora abietis-canadensis* | 1400 (PUR 61512)^a, c^ | [MPITS161-09](http://www.barcodinglife.org/index.php/MAS_DataRetrieval_OpenSequence?selectedrecordid=1082952) | *Populus grandidentata* | WI, USA | 43.024 | -89.841 | 1959 | 33.71 | 32.31 | 32.58 | UNDETM | UNDETM | UNDETM | UNDETM |
|  | 967 (QFB 25062)^a, b, c^ | [MPITS078-08](http://www.barcodinglife.org/index.php/MAS_DataRetrieval_OpenSequence?selectedrecordid=932360) | *Tsuga canadiensis* | Cap Tourmente, QC, Canada | 47.083 | -70.775 | 2008 | 33.62^d^ | 33.66^d^ | 33.43^d^ | UNDETM | UNDETM | UNDETM | UNDETM |
|  | MEA CAP1-1AT7 ^a, c^ | [PRJNA365810](https://www.ncbi.nlm.nih.gov/bioproject/365810) |  | Cap Tourmente, QC, Canada | 47.081 | -70.778 | 2009 | 31.55^d^ | 31.05^d^ | 30.57^d^ | UNDETM | UNDETM | UNDETM | UNDETM |
| *Melampsora aecidioides* | 664 (QFB 25028)^a, b, c^ | [MAITS005-08](http://www.barcodinglife.org/index.php/MAS_DataRetrieval_OpenSequence?selectedrecordid=761475) | *Populus alba* | Colquitz creek, BC | 48.459 | -123.394 | 2007 | 32.02 | 31.86^d^ | 31.31^d^ | UNDETM | UNDETM | UNDETM | UNDETM |
|  | PFH04-4 | [JN881750](https://www.ncbi.nlm.nih.gov/nuccore/JN881750) | *Populus alba* | Bouches-du-Rhône, France | 43.692 | 5.668 | 2004 | 29.66^d^ | 28.85^d^ | 28.93^d^ | n/a | n/a | n/a | n/a |
|  | PFH08-4 | [JN881753](https://www.ncbi.nlm.nih.gov/nuccore/JN881753) | *Populus* sp. | Vendée, France | 46.915 | -2.061 | 2008 | 30.52^d^ | 29.59^d^ | 29.87^d^ | n/a | n/a | n/a | n/a |
| *Melampsora allii-populina* | PFH03-23 (QFB 25064)^a, c^ | [JN881731](https://www.ncbi.nlm.nih.gov/nuccore/jN881731) | *Populus nigra* | Pazardjik, Bulgaria | 42.183 | 24.330 | 2003 | 29.76 | 29.01^d^ | 28.95^d^ | UNDETM | UNDETM | UNDETM | UNDETM |
|  | PFH04-9 (QFB 25513)^a, c^ | [JN881729](https://www.ncbi.nlm.nih.gov/nuccore/JN881729) | *Populus nigra* | Madrid, Spain | 40.452 | -3.741 | 2004 | 32.49^d^ | 32.02^d^ | 31.74^d^ | UNDETM | UNDETM | UNDETM | UNDETM |
| *Melampsora pinitorqua* | 1462 barcode^a, c^ | [JQ664560](https://www.ncbi.nlm.nih.gov/nuccore/JQ664560) | *Populus tremula* | Lot-et-Garonne, France | 44.270 | 0.027 | 1986 | 29.44^d^ | 29.40^d^ | 28.32^d^ | UNDETM | UNDETM | UNDETM | UNDETM |
|  | Mpini7 | [PRJNA190833](https://www.ncbi.nlm.nih.gov/bioproject/PRJNA190833) | n/a | Lot-et-Garonne, France | n/a | n/a | n/a | 28.70^d^ | 28.53^d^ | 27.77^d^ | n/a | n/a | UNDETM | UNDETM |
| *Melampsora hypericorum* | Mhyp1^a, c^ |  | Single aecium | Germany | n/a | n/a | 1999 | 36.89^d^ | 37.32 | 35.98^d^ | UNDETM | UNDETM | UNDETM | UNDETM |
| *Melampsora* *euphorbiae* | Meup3^a, c^ |  | Single uredium or telium | Switzerland | n/a | n/a | 1998 | 35.74^d^ | 36.75^d^ | 35.53^d^ | UNDETM | UNDETM | UNDETM | UNDETM |
| *Melampsora rostrupii* | 1282 (DAOM 127696)^a, c^ |  | *Mercurialis perrenis* | Oberhessen, Germany | 50.637 | 9.167 | 1956 | UNDETM | 36.79 | 49.19 | UNDETM | UNDETM | UNDETM | UNDETM |
|  | 1415 (TSH-R4126)^a, c^ |  | *Populus alba* | Xinjiang, China | 44.033 | 87.300 | 1984 | 37.71 | 35.86 | 35.86^d^ | UNDETM | UNDETM | UNDETM | UNDETM |
|  | PFH08-3 | [JN881752](https://www.ncbi.nlm.nih.gov/nuccore/JN881752) | *Populus alba* | Hautes-Alpes, France | 44.613 | 6.521 | 2008 | 29.65^d^ | 28.65^d^ | 28.88 | n/a | n/a | n/a | n/a |
| *Melampsora* nujiangensis | 1418 (TSH-R20046)^a^ | [MPITS179-09](http://www.barcodinglife.org/index.php/MAS_DataRetrieval_OpenSequence?selectedrecordid=1082970) | *Populus yunnanensis* | Yunnan, China | 25.980 | 98.670 | 1998 | UNDETM | 33.70 | 35.03 | UNDETM | UNDETM | UNDETM | UNDETM |
| *Melampsora magnusiana* | 1429 (GLM 77294)^a, c^ | [GQ479849](https://www.ncbi.nlm.nih.gov/nuccore/GQ479849.1) | *Corydalis cava* | Ketzerbachtal, Germany | 51.116 | 13.317 | 2005 | 29.26^d^ | 28.81^d^ | 28.58^d^ | UNDETM | UNDETM | UNDETM | UNDETM |
|  | 1328 (BPI 878595) | [JN881736](https://www.ncbi.nlm.nih.gov/nuccore/JN881736.1) | *Corydalis cava* | Burgenland, Austria | 47.767 | 16.317 | 2000 | 29.71^d^ | 29.59^d^ | 29.17^d^ | n/a | n/a | n/a | n/a |
| *Melampsora pruinosae* | 1343 (BPI 1109446) | [MPITS128-09](http://www.barcodinglife.org/index.php/MAS_DataRetrieval_OpenSequence?selectedrecordid=1082919) | *Populus diversifolia* | Xinjiang, China | 39.379 | 76.047 | 1956 | 30.36^d^ | 29.64^d^ | 29.46^d^ | n/a | n/a | n/a | n/a |
|  | 1366 (BPI 0031207) | [MPITS140-09](http://www.barcodinglife.org/index.php/MAS_DataRetrieval_OpenSequence?selectedrecordid=1082931) | *Populus pruinosae* | Bukhara Province, Uzbekistan | 39.152 | 63.610 | 1910 | 32.49^d^ | 32.39 | 32.14 | n/a | n/a | n/a | n/a |
| *Melampsora microspora* | 1407 (PUR F17540) | [JN881737](https://www.ncbi.nlm.nih.gov/nuccore/JN881737) | Populus nigra | Irak | 35.536 | 45.432 | 1950 | 31.31^d^ | 30.66^d^ | 30.60^d^ | n/a | n/a | n/a | n/a |
| *Melampsora pulcherrima* | 08ZK2 | [MEFRA013-09](http://www.barcodinglife.org/index.php/MAS_DataRetrieval_OpenSequence?selectedrecordid=1062545) | *Mercurialis annua* | Monticiano, Italy | 43.139 | 11.178 | 2008 | 40.26^d^ | 35.84^d^ | 39.64^d^ | n/a | n/a | n/a | n/a |
|  | 08ZK4 | [MEFRA015-09](http://www.barcodinglife.org/index.php/MAS_DataRetrieval_OpenSequence?selectedrecordid=1062547) | *Mercurialis annua* | Monticiano, Italy | 43.139 | 11.178 | 2008 | 39.45 | UNDETM | 42.42^d^ | n/a | n/a | n/a | n/a |
| *Melampsora epitea* | 685A | [JF825969](https://www.ncbi.nlm.nih.gov/nuccore/jf825969) | *Salix viminalis* | Svalöv, Sweden | 55.913 | 13.102 | 1996 | 21.70 | 22.50 | 28.06 | UNDETM | UNDETM | UNDETM | UNDETM |
| *Coleosporium viburni* | 808 (QFB 25209)^c^ | [COITS016-08](http://www.barcodinglife.org/index.php/MAS_DataRetrieval_OpenSequence?selectedrecordid=932245) | *Viburnum* sp. | Lac Opasatica, QC, Canada | 48.168 | -79.319 | 2007 | UNDETM | UNDETM | UNDETM | n/a | n/a | n/a | n/a |
| *Coleosporium asterum* | 237 (QFB 25087)^c^ | [COITS033-18](http://www.barcodinglife.org/index.php/MAS_DataRetrieval_OpenSequence?selectedrecordid=9502759) | *Solidago* sp. | Le Bic, QC, Canada | 48.375 | -68.695 | 2006 | UNDETM | UNDETM | UNDETM | UNDETM | UNDETM | UNDETM | UNDETM |
| *Pucciniastrum vaccinii* | 531 (DAOM 97346)^c^ | [PMITS041-08](http://www.barcodinglife.org/index.php/MAS_DataRetrieval_OpenSequence?selectedrecordid=934613) | *Vaccinium deliciosum* | Burman Lake, BC, Canada | 49.642 | -125.745 | 1961 | UNDETM | UNDETM | UNDETM | n/a | n/a | n/a | n/a |
| *Pucciniastrum americanum* | 259 barcode^c^ |  | n/a | Portneuf, QC, Canada | 46.691 | -71.888 | 1998 | UNDETM | UNDETM | UNDETM | UNDETM | UNDETM | UNDETM | UNDETM |
| *Pucciniastrum agrimoniae* | 290 (QFB 25097)^c^ | [PMITS015-08](http://www.barcodinglife.org/index.php/MAS_DataRetrieval_OpenSequence?selectedrecordid=934587) | *Agrimonia gryposepala* | Lotbinière, QC, Canada | 46.488 | -71.926 | 2005 | UNDETM | UNDETM | UNDETM | n/a | n/a | n/a | n/a |
| *Chrysomyxa ledicola* | 636 (QFB 25135)^c^ | [CHITS121-18](http://www.barcodinglife.org/index.php/MAS_DataRetrieval_OpenSequence?selectedrecordid=9462115) | *Rhododendron groenlandicum* | Zec des Martres, QC, Canada | 47.753 | -70.656 | 2007 | UNDETM | UNDETM | UNDETM | UNDETM | UNDETM | UNDETM | UNDETM |
| *Chrysomyxa pirolata* | 920 (QFB 25056)^c^ | [CHITS066-08](http://www.barcodinglife.org/index.php/MAS_DataRetrieval_OpenSequence?selectedrecordid=906818) | *Pyrola* sp. | Bic, QC, Canada | 48.359 | -68.768 | 2008 | UNDETM | UNDETM | UNDETM | n/a | n/a | n/a | n/a |
| *Cronartium ribicola* | Cr12SS2^c^ | [CRITS206-18](http://www.barcodinglife.org/index.php/MAS_DataRetrieval_OpenSequence?selectedrecordid=9462386) | Uredinial culture of mono-aecial origin | Sainte-Sophie, QC | 45.866 | -73.881 | 2012 | UNDETM | UNDETM | UNDETM | UNDETM | UNDETM | UNDETM | UNDETM |
| *Peridermium harknessii* | Alas1^c^ | [CRITS169-09](http://www.barcodinglife.org/index.php/MAS_DataRetrieval_OpenSequence?selectedrecordid=1100938) | *Pinus* sp. | AK, USA | n/a | n/a | 2006 | UNDETM | UNDETM | UNDETM | n/a | n/a | n/a | n/a |
| *Cronartium comandrae* | LP 3A^c^ |  | *Pinus banksiana* | Lac Saint-Jean, QC, Canada | 49.138 | -73.409 | 2001 | UNDETM | UNDETM | UNDETM | n/a | n/a | n/a | n/a |
| *Chrysomyxa weirii* | 574 (DAOM 172565) | [GU049473](https://www.ncbi.nlm.nih.gov/nuccore/GU049473.1) | *Picea glauca* | Riding Mtn. Nat. Park, MB, Canada | 50.805 | -100.193 | 1979 | 39.59 | UNDETM | UNDETM | UNDETM | UNDETM | UNDETM | UNDETM |
|  | 916 (QFB 25269) | [CHITS080-08](http://www.barcodinglife.org/index.php/MAS_DataRetrieval_OpenSequence?selectedrecordid=906832) | *Picea glauca* | Notre-Dame-des-Pins, Beauce, QC | 46.181 | -70.711 | 2008 | 33.26 | UNDETM | UNDETM | UNDETM | UNDETM | UNDETM | UNDETM |

UNDETM : C_t_ value = Undetermined; n/a : not available or untested.

^a^ DNA samples from target and non-target species among the Pucciniales, forming the reduced panel, used for the selection of primer pairs specific to *M. medusae*.

^b^ DNA samples from target and non-target species among the Pucciniales, forming the reduced panel, used for the selection of primer pairs specific to *M. larici-populina*.

^c^ DNA samples from target and non-target species among the Pucciniales, forming the reduced panel, used for the selection of primer pairs specific to the genus *Melampsora*.

^d^ Sequence deposited in GenBank.
