# Supplementary material for: Genome-enhanced detection and identification of fungal pathogens responsible for pine and poplar rust diseases
Source: PLoS One. 2019 Feb 6;14(2):e0210952. doi: 10.1371/journal.pone.0210952 (PMC6364900; doi:10.1371/journal.pone.0210952)
Supplement: S3 Table — (DOCX) [file pone.0210952.s004.docx]

**S3 Table. Positive detections (C_t_ values < 40.0) for *Cronartium* spp. and *Cronartium ribicola* on environmental samples.**

|  |  |  | | |  | Geographic coordinates | | CRO | | CRIB | | |
| --- | --- | --- | --- | --- | --- | --- | --- | --- | --- | --- | --- | --- |
| Sampled material | Isolate ^a^ – True positive sample | | ITS ^b^ | Provenance | | Latitude | Longitude | 30 | 46 | 65 | 146 | 190 |
| *Cronartium ribicola* associated with insects captured, using Plexiglas boards coated with the *Tree Tangle Foot* glue, in a white pine plantation diseased with blister rust, in 1997 ^a^ | 102 *Megaselia nigriceps* (Diptera : Phoridae) | | Y | Saint-Cyprien, QC, Canada | | 46.377 | -70.229 | 33.43 | 33.52 | 35.35 | 33.94 | 41.10 |
|  | 103 (Diptera : Phoridae) | | Y | Saint-Cyprien, QC, Canada | | 46.377 | -70.229 | 35.03 ^c^ | 35.69 | 41.32 | 35.77 | 41.02 ^c^ |
|  | 161 *Megaselia arcticae* (Diptera : Phoridae) | | Y | Saint-Cyprien, QC, Canada | | 46.377 | -70.229 | 34.83 | 35.97 | 36.16 | UNDETM | 41.09 ^c^ |
|  | 167 *Megaselia arcticae* (Diptera : Phoridae) | | Y | Saint-Cyprien, QC, Canada | | 46.377 | -70.229 | 34.62 | 33.68 | 35.22 | 32.92 | 41.46 ^c^ |
|  | 171 *Megaselia arcticae* (Diptera : Phoridae) | | Y | Saint-Cyprien, QC, Canada | | 46.377 | -70.229 | 35.25 | 35.44 | 35.62 | 36.07 | 39.67 ^c^ |
|  | 173 (Diptera : Cecidomyiidae) | | Y | Saint-Cyprien, QC, Canada | | 46.377 | -70.229 | 36.05 ^c^ | 47.69 | 35.91 | 34.76 | 41.19 ^c^ |
|  | 206 *Megaselia* sp. (Diptera : Phoridae) | | Y | Saint-Cyprien, QC, Canada | | 46.377 | -70.229 | 30.79 | 29.64 | 31.92 | 29.64 | 34.63 |
|  | 207 *Megaselia* sp. (Diptera : Phoridae) | | Y | Saint-Cyprien, QC, Canada | | 46.377 | -70.229 | 31.63 | 29.56 | 31.43 | 30.40 | 36.85 |
|  | 208 *Megaselia* sp. (Diptera : Phoridae) | | Y | Saint-Cyprien, QC, Canada | | 46.377 | -70.229 | UNDETM | 34.65 | 35.74 | 35.14 | 40.61 |
|  | n = 9 | |  |  | |  |  | 8 (88.9%) | 8 (88.9%) | 8 (88.9%) | 8 (88.9%) | 3 (33.3%) |
| White pine needles artificially inoculated by *Cronartium ribicola* | 608i 219-11 | | Y | Île d’Orléans, QC, Canada | | 46.877 | -71.094 | 23.96 | 23.07 | 23.93 | 23.76 | 27.73 |
|  | 608i 219-12 | | Y | Île d’Orléans, QC, Canada | | 46.877 | -71.094 | 34.93 | 33.19 | 34.07 | 34.43 | 43.36 |
|  | 608i 219-2 | | Y | Île d’Orléans, QC, Canada | | 46.877 | -71.094 | 30.15 | 29.70 | 30.53 | 30.11 | 35.20 |
|  | 608i 219-3 | | Y | Île d’Orléans, QC, Canada | | 46.877 | -71.094 | 33.88 | 33.27 | 34.53 | 32.99 | 41.31 |
|  | 608i 220-9 | | Y | Île d’Orléans, QC, Canada | | 46.877 | -71.094 | 23.88 | 23.51 | 24.76 | 24.16 | 27.86 |
|  | 608i 221-1 | | Y | Île d’Orléans, QC, Canada | | 46.877 | -71.094 | 29.25 | 28.13 | 29.47 | 28.79 | 33.51 |
|  | 608i 221-10 | | Y | Île d’Orléans, QC, Canada | | 46.877 | -71.094 | 30.14 | 28.70 | 29.90 | 29.02 | 34.14 |
|  | 608i 221-13 | | Y | Île d’Orléans, QC, Canada | | 46.877 | -71.094 | 30.93 | 29.54 | 30.56 | 30.24 | 35.15 |
|  | 608i 221-3 | | Y | Île d’Orléans, QC, Canada | | 46.877 | -71.094 | 25.77 | 25.01 | 26.01 | 25.57 | 30.21 |
|  | 608i 221-5 | | Y | Île d’Orléans, QC, Canada | | 46.877 | -71.094 | 33.14 | 32.88 | 34.42 | 33.86 | 38.08 |
|  | 608i 224-5 | | Y | Île d’Orléans, QC, Canada | | 46.877 | -71.094 | 27.62 | 27.40 | 28.56 | 27.86 | 31.19 |
|  | 608i 224-7 | | Y | Île d’Orléans, QC, Canada | | 46.877 | -71.094 | 31.84 | 31.10 | 31.82 | 31.79 | 36.81 |
|  | 608i 225-11 | | Y | Île d’Orléans, QC, Canada | | 46.877 | -71.094 | 29.20 | 28.74 | 29.50 | 29.43 | 34.17 |
|  | 608i 226-4 | | Y | Île d’Orléans, QC, Canada | | 46.877 | -71.094 | 31.60 | 30.24 | 31.82 | 31.24 | 36.31 |
|  | 608i 230-11 | | Y | Île d’Orléans, QC, Canada | | 46.877 | -71.094 | 32.40 | 31.90 | 33.47 | 32.36 | 37.57 |
|  | 608i 230-4 | | Y | Île d’Orléans, QC, Canada | | 46.877 | -71.094 | 30.85 | 28.99 | 30.08 | 29.74 | 34.29 |
|  | 608i 232-4 | | Y | Île d’Orléans, QC, Canada | | 46.877 | -71.094 | 34.70 | 33.22 | 34.44 | 36.63 | 40.17 |
|  | 608i 236-2 | | Y | Île d’Orléans, QC, Canada | | 46.877 | -71.094 | 29.78 | 28.63 | 29.51 | 29.43 | 34.11 |
|  | 608i 236-5 | | Y | Île d’Orléans, QC, Canada | | 46.877 | -71.094 | 27.78 | 26.98 | 28.26 | 28.04 | 32.25 |
|  | 608i 236-8 | | Y | Île d’Orléans, QC, Canada | | 46.877 | -71.094 | 31.50 | 30.48 | 31.58 | 31.35 | 37.37 |
|  | 608i 237-3 | | Y | Île d’Orléans, QC, Canada | | 46.877 | -71.094 | 31.14 | 30.52 | 31.11 | 30.67 | 35.87 |
|  | 608i 237-6 | | Y | Île d’Orléans, QC, Canada | | 46.877 | -71.094 | 30.11 | 29.44 | 30.25 | 30.00 | 35.07 |
|  | 608i 238-1 | | Y | Île d’Orléans, QC, Canada | | 46.877 | -71.094 | 30.97 | 30.69 | 32.07 | 31.33 | 37.04 |
|  | n = 23 | |  |  | |  |  | 23 (100.0%) | 23 (100.0%) | 23 (100.0%) | 23 (100.0%) | 20 (87.0%) |
| White pine stems with symptoms of infection with *Cronartium ribicola* sampled in nurseries | S- 62528-1 | | n/a | Saint-Modeste, QC, Canada | | 47.839 | -69.392 | 27.49 | 27.44 | 21.34 | 26.68 | 32.13 |
|  | S- 62528-2 | | n/a | Saint-Modeste, QC, Canada | | 47.839 | -69.392 | 27.07 | 25.61 | 27.10 | 26.94 | 32.28 |
|  | S- 62529-1 | | n/a | Saint-Modeste, QC, Canada | | 47.839 | -69.392 | 28.10 | 27.62 | 23.85 | 26.15 | 33.26 |
|  | 76742 | | n/a | Saint-Modeste, QC, Canada | | 47.839 | -69.392 | 25.74 | 25.25 | 26.14 | 25.15 | 29.64 |
|  | 76744 | | n/a | Saint-Modeste, QC, Canada | | 47.839 | -69.392 | 24.54 | 24.08 | 25.05 | 24.42 | 28.36 |
|  | 76749 | | n/a | Saint-Modeste, QC, Canada | | 47.839 | -69.392 | 35.48 | 34.12 | 36.22 | 36.42 | UNDETM |
|  | 76750 | | n/a | Saint-Modeste, QC, Canada | | 47.839 | -69.392 | 23.10 | 22.75 | 23.73 | 23.19 | 27.09 |
|  | 76741 | | n/a | Saint-Modeste, QC, Canada | | 47.839 | -69.392 | 24.40 | 23.76 | 24.76 | 24.44 | 27.90 |
|  | 76604 | | n/a | Sainte-Luce, QC, Canada | | 48.515 | -68.373 | 26.59 | 26.05 | 27.09 | 26.66 | 30.48 |
|  | 76743 | | n/a | Saint-Modeste, QC, Canada | | 47.839 | -69.392 | 23.82 | 23.37 | 24.15 | 23.78 | 27.28 |
|  | 76752 | | n/a | Saint-Modeste, QC, Canada | | 47.839 | -69.392 | 23.67 | 23.22 | 24.29 | 23.77 | 27.25 |
|  | n = 11 | |  |  | |  |  | 11 (100.0%) | 11 (100.0%) | 11 (100.0%) | 11 (100.0%) | 10 (90.9%) |
| Blackcurrant leaves naturally infected with *Cronartium ribicola* | QC-1 | | Y | Val-Brillant, QC, Canada | | 48.527 | -67.561 | 25.64^d^ | 24.88 | 26.07 | 25.85 | 29.49 |
|  | QC-2 | | Y | Val-Brillant, QC, Canada | | 48.527 | -67.561 | 29.45^d^ | 28.90 | 29.95 | 29.67 | 33.81 |
|  | QC-4 | | Y | Saint-André d'Argenteuil, QC | | 45.601 | -74.346 | 29.53 | 28.55 | 29.77 | 29.38 | 33.65 |
|  | NS-1 | | Y | Truro, NS, Canada | | 45.388 | -63.257 | 26.88^d^ | 26.33 | 26.98 | 26.65 | 30.32 |
|  | NB-1 | | Y | Hillsborough, NB, Canada | | 45.917 | -64.638 | 25.53^d^ | 24.66 | 25.71 | 25.19 | 28.45 |
|  | PEI-1 | | Y | Hunter River, PEI, Canada | | 46.336 | -63.367 | 26.69^d^ | 25.96 | 26.98 | 26.61 | 30.65 |
|  | PEI-2 | | Y | Hunter River, PEI, Canada | | 46.336 | -63.367 | 35.51 | 36.06 | 36.80 | 36.24 | 45.49 |
|  | PEI-3 | | Y | Kensington, PEI, Canada | | 46.393 | -63.522 | 26.26^d^ | 25.54 | 26.72 | 26.30 | 30.35 |
|  | n = 8 | |  |  | |  |  | 8 (100.0%) | 8 (100.0%) | 8 (100.0%) | 8 (100.0%) | 7 (87.5%) |
| Single telia sampled from blackcurrant leaves naturally infected with *Cronartium ribicola* | Telium-8 | | Y | Lethbridge, AB, Canada | | 49.699 | -112.713 | 31.12 | 29.80 | 30.88 | 30.56 | 35.59 |
|  | Telium-10 | | Y | Lethbridge, AB, Canada | | 49.699 | -112.713 | UNDETM | 28.83 | 30.27 | 29.64 | 33.02 |
|  | Telium-18 | | n/a | Lethbridge, AB, Canada | | 49.691 | -112.842 | 33.23 | 32.66 | 34.48 | 33.73 | 38.93 |
|  | Telium-20 | | Y | Lethbridge, AB, Canada | | 49.691 | -112.842 | 29.92 | 29.06 | 30.20 | 29.57 | 34.71 |
|  | Telium-28 | | Y | AB, Canada | | 49.700 | -112.714 | 31.74 | 30.87 | 32.04 | 31.33 | 36.18 |
|  | Telium-30 | | Y | AB, Canada | | 49.700 | -112.714 | 29.71 | 28.93 | 30.12 | 29.57 | 34.18 |
|  | Telium-38 | | n/a | Fort MacLeod, AB, Canada | | 49.802 | -113.409 | 31.52 | 30.76 | 31.81 | 31.12 | 36.42 |
|  | Telium-40 | | Y | Fort MacLeod, AB, Canada | | 49.802 | -113.409 | 30.44 | 29.70 | 30.54 | 30.33 | 35.49 |
|  | Telium-48 | | Y | Brooks, AB, Canada | | 50.552 | -111.851 | 29.70 | 29.32 | 30.21 | 29.83 | 34.96 |
|  | Telium-50 | | Y | Brooks, AB, Canada | | 50.552 | -111.851 | 30.37 | 29.28 | 30.77 | 30.19 | 34.71 |
|  | Telium-58 | | Y | Lethbridge, AB, Canada | | 49.691 | -112.842 | 30.03 | 29.11 | 30.65 | 30.25 | 35.16 |
|  | Telium-60 | | Y | Lethbridge, AB, Canada | | 49.691 | -112.842 | 31.10 | 30.22 | 31.07 | 30.62 | 36.67 |
|  | Telium-68 | | Y | Lethbridge, AB, Canada | | 49.691 | -112.842 | 31.12 | 30.43 | 32.24 | 30.99 | 36.20 |
|  | Telium-70 | | Y | Lethbridge, AB, Canada | | 49.691 | -112.842 | 32.56 | 31.90 | 33.10 | 32.02 | 37.18 |
|  | n = 14 | |  |  | |  |  | 13 (92.9%) | 14 (100.0%) | 14 (100.0%) | 14 (100.0%) | 14 (100.0%) |

UNDETM : C_t_ value = Undetermined; n/a : untested.

^a^ Insect identification, based on COI sequence, follows the name of samples.

^b^ DNA barcoding identification of *C. ribicola*, based on ITS sequence.

^c^ means that one of the technical replicates = UNDETM.

^d^ Sequence deposited in GenBank.
