# Supplementary material for: Genome-enhanced detection and identification of fungal pathogens responsible for pine and poplar rust diseases
Source: PLoS One. 2019 Feb 6;14(2):e0210952. doi: 10.1371/journal.pone.0210952 (PMC6364900; doi:10.1371/journal.pone.0210952)
Supplement: S4 Table — (DOCX) [file pone.0210952.s005.docx]

**S4 Table. Positive detections (C_t_ values < 40.0) for *Melampsora* spp., *Melampsora medusae* f. sp. *deltoidae* and *Melampsora larici-populina* on environmental samples.**

|  |  |  |  | Geographic coordinates | | MEL | | | MM | | MLP | |
| --- | --- | --- | --- | --- | --- | --- | --- | --- | --- | --- | --- | --- |
| Sampled material | Isolate – True positive sample | ITS^a^ | Provenance | Latitude | Longitude | 40 | 100 | 176 | 53 | 74 | 104 | 133 |
| Larch needles naturally infected with *Melampsora medusae* f. sp. *deltoidae* and/or *Melampsora larici-populina* | LOTB-MS01-m (01) | Y | Lotbinière, QC, Canada | 46.488 | -71.926 | 32.01 | 30.97 | 31.28 | 36.43 | 32.65 | UNDETM | UNDETM |
|  | LOTB-MS04-m (04) | Y | Lotbinière, QC, Canada | 46.488 | -71.926 | 32.46 | 31.63 | 30.83 | 30.19 | 32.30 | UNDETM | UNDETM |
|  | LOTB-MS07-k (07) | Y | Lotbinière, QC, Canada | 46.488 | -71.926 | 32.92 | 32.27 | 31.96 | 31.38 | 32.71 | UNDETM | UNDETM |
|  | LOTB-MS08-k (08) | Y | Lotbinière, QC, Canada | 46.488 | -71.926 | 32.38 | 32.42 | 31.80 | 31.17 | 32.84 | UNDETM | UNDETM |
|  | CR-POP-5 (Ec) (05) | Y | Cap Rouge, QC, Canada | 46.765 | -71.355 | 32.26 | 31.62 | 31.96 | 31.84 | 33.13 | UNDETM | UNDETM |
|  | CR-POP-10 (Ec) (10) | Y | Cap Rouge, QC, Canada | 46.765 | -71.355 | 29.33 | 28.35 | 28.61 | 29.54 | 29.60 | UNDETM | UNDETM |
|  | CR-CHAMP-15 (Ec) (15) | Y | Cap Rouge, QC, Canada | 46.765 | -71.355 | 32.96 | 32.10 | 31.70 | 31.90 | 33.52 | UNDETM | UNDETM |
|  | CR-CHAMP-20 (Ec) (20) | Y | Cap Rouge, QC, Canada | 46.765 | -71.355 | 32.09 | 31.67 | 31.33 | 30.62 | 32.97 | UNDETM | UNDETM |
|  | CR-STOP-24 (Ec) (24) | Y | Cap Rouge, QC, Canada | 46.765 | -71.355 | 33.01 | 32.37 | 32.15 | 31.53 | 33.59 | UNDETM | UNDETM |
|  | CR-STOP-29 (Ec) (29) | Y | Cap Rouge, QC, Canada | 46.765 | -71.355 | 32.20 | 31.94 | 32.30 | UNDETM | 32.95 | UNDETM | UNDETM |
|  | CR-J1-36 (Ec) (36) | Y | Cap Rouge, QC, Canada | 46.765 | -71.355 | 32.12 | 31.28 | 31.38 | 31.68 | 32.41 | UNDETM | UNDETM |
|  | 04-SF-Ec-12 | n/a | Saint-Félicien, QC, Canada | 48.650 | -72.449 | 32.96 | 33.18 | 31.98 | 32.99 | 38.54 | UNDETM | UNDETM |
|  | 04-SF-Ec-14 | n/a | Saint-Félicien, QC, Canada | 48.650 | -72.449 | 29.53 | 29.04 | 28.62 | 34.02 | 29.77 | UNDETM | UNDETM |
|  | 04-SF-Ec-16 | Y | Saint-Félicien, QC, Canada | 48.650 | -72.449 | 30.98 | 30.41 | 30.68 | 32.65 | 31.25 | UNDETM | UNDETM |
|  | 04-SF-Ec-20 | Y | Saint-Félicien, QC, Canada | 48.650 | -72.449 | 27.08 | 26.60 | 26.33 | 27.32 | 27.74 | UNDETM | UNDETM |
|  | 04-LOTB-Ec-03 | Y | Lotbinière, QC, Canada | 46.488 | -71.926 | 34.47 | 32.60 | 33.48 | UNDETM | UNDETM | 31.64 | 32.98 |
|  | 04-LOTB-Ec-11 | Y | Lotbinière, QC, Canada | 46.488 | -71.926 | 34.42 | 32.25 | 32.69 | UNDETM | UNDETM | 33.08 | 32.79 |
|  | 04-LOTB-Ec-14 | Y | Lotbinière, QC, Canada | 46.488 | -71.926 | 34.39 | 31.79 | 31.96 | UNDETM | UNDETM | 32.51 | 32.91 |
|  | 04-LOTB-Ec-15 | Y | Lotbinière, QC, Canada | 46.488 | -71.926 | 35.37 | 33.07 | 33.48 | UNDETM | UNDETM | 33.88 | 34.42 |
|  | n = 19 |  |  |  |  | 19 (100.0%) | 19 (100.0%) | 19 (100.0%) | 14 (73.7%) | 15 (78.9%) | 4 (21.1%) | 4 (21.1%) |
| Hybrid poplar leaves naturally infected with *Melampsora medusae* f. sp. *deltoidae* and/or *Melampsora larici-populina* | LOTB 01-3333-1B (1-8) | Y | Lotbinière, QC, Canada | 46.488 | -71.926 | 26.92 | 26.68 | 26.11 | 26.03 | 27.51 | 26.12 | 27.95 |
|  | LOTB 04-3333-1A (1-55) | Y | Lotbinière, QC, Canada | 46.488 | -71.926 | 28.88 | 27.88 | 27.27 | 30.98 | 32.98 | 26.16 | 27.77 |
|  | LOTB 07-3333-3A (2-53) | Y | Lotbinière, QC, Canada | 46.488 | -71.926 | 25.90 | 25.36 | 24.80 | 24.19 | 26.72 | 28.12 | 29.21 |
|  | LOTB 01-3342-1A (1-43) | Y | Lotbinière, QC, Canada | 46.488 | -71.926 | 28.07 | 27.93 | 27.22 | 27.36 | 29.61 | 29.38 | 30.24 |
|  | LOTB 04-3342-3A (1-65) | Y | Lotbinière, QC, Canada | 46.488 | -71.926 | 26.28 | 25.09 | 24.74 | 28.32 | 30.07 | 23.95 | 25.30 |
|  | LOTB 07-3342-1A (2-55) | Y | Lotbinière, QC, Canada | 46.488 | -71.926 | 24.60 | 24.04 | 23.67 | 23.41 | 25.57 | 26.84 | 28.39 |
|  | LOTB 07-3342-2A (2-57) | Y | Lotbinière, QC, Canada | 46.488 | -71.926 | 27.54 | 26.65 | 26.12 | 26.61 | 28.89 | 26.52 | 27.99 |
|  | LOTB 01-3675-1A (1-1) | Y | Lotbinière, QC, Canada | 46.488 | -71.926 | 30.56 | 29.74 | 29.30 | 28.32 | 30.88 | 36.70^b^ | 38.20^b^ |
|  | LOTB 01-4723-1A (1-37) | Y | Lotbinière, QC, Canada | 46.488 | -71.926 | 27.22 | 26.71 | 24.88 | 24.83 | 26.98 | 35.64 | 36.42 |
|  | LOTB 01-136652-1A (1-13) | Y | Lotbinière, QC, Canada | 46.488 | -71.926 | 28.75 | 28.78 | 28.05 | 27.05 | 29.68 | 33.39 | 33.94 |
|  | LOTB 01-136652-2A (1-15) | Y | Lotbinière, QC, Canada | 46.488 | -71.926 | 31.50 | 30.31 | 29.81 | 36.66 | 37.36 | 28.58 | 29.93 |
|  | LOTB 04-136652-1A (1-91) | Y | Lotbinière, QC, Canada | 46.488 | -71.926 | 28.73 | 28.17 | 27.81 | 27.57 | 29.40 | 28.59 | 29.86 |
|  | LOTB 07-136652-3B (2-30) | Y | Lotbinière, QC, Canada | 46.488 | -71.926 | 29.52 | 29.20 | 28.38 | 27.89 | 29.77 | 32.22 | 32.65 |
|  | LOTB 04-136703-1B (1-86) | Y | Lotbinière, QC, Canada | 46.488 | -71.926 | 24.39 | 23.23 | 23.33 | 25.16 | 26.91 | 22.61 | 24.28 |
|  | LOTB 07-136703-1A (2-43) | Y | Lotbinière, QC, Canada | 46.488 | -71.926 | 27.38 | 25.99 | 25.67 | 31.85 | 34.06 | 24.49 | 26.08 |
|  | LOTB 01-136751-3B (1-54) | Y | Lotbinière, QC, Canada | 46.488 | -71.926 | 27.34 | 25.85 | 25.52 | 34.07 | 37.35 | 24.16 | 25.75 |
|  | LOTB 08-136751-1A (2-19) | Y | Lotbinière, QC, Canada | 46.488 | -71.926 | 27.28 | 26.01 | 25.28 | 36.31^b^ | 37.27^b^ | 24.02 | 25.73 |
|  | LOTB 01-162682-3B (1-36) | Y | Lotbinière, QC, Canada | 46.488 | -71.926 | 26.26 | 26.56 | 25.54 | 24.61 | 26.76 | 36.20^b^ | UNDETM |
|  | LOTB 04-162682-3B (1-72) | Y | Lotbinière, QC, Canada | 46.488 | -71.926 | 25.48 | 25.18 | 24.67 | 23.77 | 26.14 | 33.04 | 34.07 |
|  | LOTB 07-162682-3A (2-17) | Y | Lotbinière, QC, Canada | 46.488 | -71.926 | 29.10 | 28.89 | 28.18 | 27.60 | 29.35 | 35.71 | 37.71 |
|  | LOTB 01-915313-1B (1-20) | Y | Lotbinière, QC, Canada | 46.488 | -71.926 | 27.22 | 25.70 | 25.40 | 28.81 | 31.69 | 24.80 | 25.70 |
|  | LOTB 04-915313-1A (2-1) | Y | Lotbinière, QC, Canada | 46.488 | -71.926 | 33.86 | 32.54 | 31.73 | 34.32 | 38.34 | 31.11 | 32.82 |
|  | LOTB 04-915313-1B (2-2) | Y | Lotbinière, QC, Canada | 46.488 | -71.926 | 31.91 | 30.88 | 29.90 | 34.28 | 38.33 | 29.21 | 30.57 |
|  | St-Modeste 18451-A (2-67) | Y | Saint-Modeste, QC, Canada | 47.839 | -69.392 | 27.29 | 25.57 | 25.43 | 35.49 | 38.28 | 24.84 | 26.17 |
|  | St-Modeste 18451-B (2-68) | Y | Saint-Modeste, QC, Canada | 47.839 | -69.392 | 26.11 | 24.16 | 24.16 | 31.60 | 33.62 | 23.72 | 24.86 |
|  | St-Modeste 18454-A (2-69) | Y | Saint-Modeste, QC, Canada | 47.839 | -69.392 | 33.49 | 32.52 | 32.87 | 32.51 | 33.76 | 34.24 | 35.34 |
|  | St-Modeste 18454-B (2-70) | Y | Saint-Modeste, QC, Canada | 47.839 | -69.392 | 35.68 | 35.09 | 34.97 | UNDETM | UNDETM | 33.32 | 34.45 |
|  | St-Modeste 18455-A (2-71) | Y | Saint-Modeste, QC, Canada | 47.839 | -69.392 | 27.37 | 25.33 | 25.46 | 33.51 | 36.85 | 24.99 | 26.13 |
|  | St-Modeste 18455-B (2-72) | Y | Saint-Modeste, QC, Canada | 47.839 | -69.392 | 26.26 | 24.59 | 24.30 | 34.66 | 38.46 | 23.79 | 25.05 |
|  | Ste-Luce 18716-B (2-73) | Y | Sainte-Luce, QC, Canada | 48.515 | -68.373 | 26.99 | 24.86 | 24.89 | 34.09 | 35.93 | 24.12 | 25.52 |
|  | Ste-Luce 18716-A (2-74) | Y | Sainte-Luce, QC, Canada | 48.515 | -68.373 | 27.42 | 25.44 | 25.20 | 35.39 | 36.49 | 24.79 | 25.97 |
|  | Taché 18707-A (2-75) | Y | Témiscouata-sur-le-Lac, QC | 47.666 | -68.869 | 26.00 | 25.38 | 25.18 | 24.42 | 26.72 | 35.93 | 35.30 |
|  | Taché 18707-B (2-76) | Y | Témiscouata-sur-le-Lac, QC | 47.666 | -68.869 | 25.08 | 24.13 | 24.13 | 23.26 | 25.45 | 28.12 | 29.83 |
|  | n = 33 |  |  |  |  | 33 (100.0%) | 33 (100.0%) | 33 (100.0%) | 32 (97.0%) | 32 (97.0%) | 33 (100.0%) | 32 (97.0%) |

UNDETM : C_t_ value = Undetermined; n/a : untested.

^a^ Identification of *M. medusae* f. sp. *deltoidae* and/or *M. larici-populina*, based on real-time PCR assays using species-specific primers targeting ITS region [38].

^b^ means that one of the technical replicates = UNDETM.
