# Supplementary material for: Genome-enhanced detection and identification of fungal pathogens responsible for pine and poplar rust diseases
Source: PLoS One. 2019 Feb 6;14(2):e0210952. doi: 10.1371/journal.pone.0210952 (PMC6364900; doi:10.1371/journal.pone.0210952)
Supplement: S5 Table — Values indicate the number of positive (+) and negative (-) samples per species that were tested for each targeted taxonomic group. (DOCX) [file pone.0210952.s006.docx]

**S5 Table. Species samples used for testing/interpretation of the assays.** Values indicate the number of positive (+) and negative (-) samples per species that were tested for each targeted taxonomic group.

|  | | | *Cronartium* spp. | |  | *C. ribicola* | |  | *Melampsora* spp. | |  | MLP^a^ | |  | MM^a^ | |
| --- | --- | --- | --- | --- | --- | --- | --- | --- | --- | --- | --- | --- | --- | --- | --- | --- |
|  | | | + | - |  | + | - |  | + | - |  | + | - |  | + | - |
| *Cronartium*/*Endocronartium* | | |  |  |  |  |  |  |  |  |  |  |  |  |  |  |
|  | | *C. ribicola* | 20 | - |  | 64 | - |  | - | 1 |  | - | 1 |  | - | 1 |
|  | | *C. comandrae* | 3 | - |  | - | 3 |  | - | 1 |  | - | - |  | - | - |
|  | | *C. quercuum* f. sp. *fusiforme* | 3 | - |  | - | 3 |  | - | - |  | - | - |  | - | - |
|  | | *C. quercuum* f. sp. *banksianae* | 3 | - |  | - | 3 |  | - | - |  | - | - |  | - | - |
|  | | *C. quercuum* f. sp. *virginianae* | 1 | - |  | - | 1 |  | - | - |  | - | - |  | - | - |
|  | | *C. comptoniae* | 1 | - |  | - | 1 |  | - | - |  | - | - |  | - | - |
|  | | *C. flaccidum* | 1 | - |  | - | 1 |  | - | - |  | - | - |  | - | - |
|  | | *C. strobilinum* | 1 | - |  | - | 1 |  | - | - |  | - | - |  | - | - |
|  | | *C. coleosporioides* | 1 | - |  | - | 1 |  | - | - |  | - | - |  | - | - |
|  | | *Cronartium* sp. on *P. armandii* | 3 | - |  | - | 3 |  | - | - |  | - | - |  | - | - |
|  | | *E. harknessii* | 3 | - |  | - | 3 |  | - | 1 |  | - | - |  | - | - |
|  | | *E. pini* | 1 | - |  | - | 1 |  | - | - |  | - | - |  | - | - |
| *Melampsora* | | |  |  |  |  |  |  |  |  |  |  |  |  |  |  |
|  | *M. medusae*^b^ | | - | 1 |  | - | 1 |  | 21 | - |  | - | 18 |  | 29 | - |
|  | *M. occidentalis* | | - | 1 |  | - | - |  | 1 | - |  | - | 1 |  | - | 1 |
|  | *M.* x*columbiana* | | - |  |  | - | - |  | 2 | - |  | - | 1 |  | 6 | - |
|  | *M. abietis-canadensis* | | - | 1 |  | - | - |  | 3 | - |  | - | 3 |  | - | 3 |
|  | *M. larici-populina* | | - | 1 |  | - | 1 |  | 24 | - |  | 59 | - |  | - | 7 |
|  | *M. allii-populina* | | - | 1 |  | - | - |  | 2 | - |  | - | 2 |  | - | 2 |
|  | *M. aecidioides* | | - | 1 |  | - | - |  | 3 | - |  | - | 1 |  | - | 1 |
|  | *M. pinitorqua* | | - | 1 |  | - | - |  | 2 | - |  | - | 2 |  | - | 1 |
|  | *M. epitea* | | - | 1 |  | - | - |  | - | - |  | - | - |  | - | - |
|  | *M. hypericorum* | | - | - |  | - | - |  | 1 | - |  | - | 1 |  | - | 1 |
|  | *M. euphorbiae* | | - | - |  | - | - |  | 1 | - |  | - | 1 |  | - | 1 |
|  | *M. larici-tremulae* | | - | - |  | - | - |  | 1 | - |  | - | 1 |  | - | 1 |
|  | *M. rostrupii* | | - | - |  | - | - |  | 3 | - |  | - | 2 |  | - | 2 |
|  | *M. nujiangensis* | | - | - |  | - | - |  | 1 | - |  | - | 1 |  | - | 1 |
|  | *M. magnusiana* | | - | - |  | - | - |  | 2 | - |  | - | 1 |  | - | 1 |
|  | *M. pruinosae* | | - | - |  | - | - |  | 2 | - |  | - | - |  | - | - |
|  | *M. microspora* | | - | - |  | - | - |  | 1 | - |  | - | - |  | - | - |
|  | *M. pulcherrima* | | - | - |  | - | - |  | 2 | - |  | - | - |  | - | - |
|  | MLP + MMD^c^ | | - | - |  | - | - |  | 32 | - |  | 32 | - |  | 32 | - |
| *Coleosporium* | | |  |  |  |  |  |  |  |  |  |  |  |  |  |  |
|  | | *C. viburni* | - | 1 |  | - | 1 |  | - | 1 |  | - | - |  | - | - |
|  | | *C. asterum* | - | 1 |  | - | 1 |  | - | 1 |  | - | 1 |  | - | 1 |
|  | | *C. pinicola* | - | 1 |  | - | - |  | - | - |  | - | - |  | - | - |
|  | | *C. tussilaginis* | - | 1 |  | - | 1 |  | - | - |  | - | - |  | - | - |
| *Pucciniastrum* | | |  |  |  |  |  |  |  |  |  |  |  |  |  |  |
|  | | *P. vaccinii* | - | 1 |  | - | 1 |  | - | 1 |  | - | - |  | - | - |
|  | | *P. goeppertianum* | - | 1 |  | - | - |  | - | - |  | - | - |  | - | - |
|  | | *P. americanum* | - | 1 |  | - | 1 |  | - | 1 |  | - | 1 |  | - | 1 |
|  | | *P. epilobii* | - | 1 |  | - | - |  | - | - |  | - | - |  | - | - |
|  | | *P. agrimoniae* | - | 1 |  | - | 1 |  | - | 1 |  | - | - |  | - | - |
| *Chrysomyxa* | | |  |  |  |  |  |  |  |  |  |  |  |  |  |  |
|  | | *C. cassandrae* | - | 1 |  | - | - |  | - | - |  | - | - |  | - | - |
|  | | *C. chiogenis* | - | 1 |  | - | - |  | - | - |  | - | - |  | - | - |
|  | | *C. weirii* | - | 1 |  | - | - |  | - | 2 |  | - | 1 |  | - | - |
|  | | *C. ledicola* | - | 1 |  | - | 1 |  | - | 1 |  | - | 1 |  | - | 1 |
|  | | *C. woroninii* | - | 1 |  | - | - |  | - | - |  | - | - |  | - | - |
|  | | *C. empetri* | - | 1 |  | - | - |  | - | - |  | - | - |  | - | - |
|  | | *C. pirolata* | - | 1 |  | - | 1 |  | - | 1 |  | - | - |  | - | - |
| Other negative samples | | | - | - |  | *-* | - |  | - | - |  | - | 3 |  | - | 4 |

^a^MLP: *Melampsora larici-populina*; MM: *M. medusae*.

^b^Includes the two *formae speciales* of *M. medusae*: *M. medusae* f. sp. *deltoidae* or *M. medusae* f. sp. *tremuloidae*.

^c^Mix between *M. larici-populina* and *M. medusae* f. sp. *deltoidae*.
