# Supplementary material for: Genome-enhanced detection and identification of fungal pathogens responsible for pine and poplar rust diseases
Source: PLoS One. 2019 Feb 6;14(2):e0210952. doi: 10.1371/journal.pone.0210952 (PMC6364900; doi:10.1371/journal.pone.0210952)
Supplement: S6 Table — The replicate numbers in the first column correspond to the three independent extractions from the same number of spores. (DOCX) [file pone.0210952.s007.docx]

**S6 Table. Detection limit of the *Cronartium* genus- and *Cronartium ribicola*-specific assays, using a known amount of *Cronartium ribicola* aeciospores from which DNA was extracted.** The replicate numbers in the first column correspond to the three independent extractions from the same number of spores.

| No. of spores  per reaction | Means of C_t_ values calculated from three technical replicates (± SD) | | | | |
| --- | --- | --- | --- | --- | --- |
|  | CRO30 | CRO46 | CRIB65 | CRIB146 | CRIB190 |
| 30 000 | 19.91 (0.18) | 18.94 (0.19) | 20.14 (0.08) | 19.50 (0.12) | 23.22 (0.15) |
| 30 000 | 19.79 (0.11) | 18.81 (0.12) | 20.08 (0.13) | 19.39 (0.13) | 23.13 (0.10) |
| 30 000 | 19.84 (0.07) | 18.77 (0.02) | 20.12 (0.05) | 19.52 (0.08) | 23.01 (0.28) |
| 500 | 25.73 (0.10) | 25.45 (0.12) | 26.67 (0.16) | 26.10 (0.13) | 29.74 (0.24) |
| 500 | 25.51 (0.08) | 25.40 (0.03) | 26.67 (0.10) | 26.07 (0.03) | 29.09 (0.07) |
| 500 | 25.42 (0.12) | 25.20 (0.05) | 26.59 (0.07) | 25.92 (0.02) | 29.13 (0.15) |
| 100 | 28.31 (0.06) | 27.51 (0.16) | 28.46 (0.08) | 28.24 (0.22) | 32.66 (0.25) |
| 100 | 27.97 (0.01) | 27.01 (0.08) | 28.29 (0.08) | 27.73 (0.12) | 32.03 (0.06) |
| 100 | 28.39 (0.16) | 27.71 (0.18) | 28.54 (0.09) | 27.95 (0.10) | 33.05 (0.34) |
| 25 | 30.39 (0.11) | 29.76 (0.11) | 30.57 (0.21) | 30.74 (0.22) | 35.43 (0.69) |
| 25 | 30.48 (0.42) | 29.63 (0.12) | 30.64 (0.22) | 30.35 (0.11) | 34.85 (0.30) |
| 25 | 30.37 (0.41) | 29.81 (0.27) | 30.97 (0.03) | 30.44 (0.22) | 34.47 (0.38) |
| 10 | 31.28 (0.36) | 30.49 (0.34) | 31.68 (0.22) | 31.13 (0.09) | 35.40 (0.30) |
| 10 | 31.80 (0.12) | 31.00 (0.63) | 32.23 (0.51) | 31.56 (0.22) | 36.17 (0.40) |
| 10 | 31.37 (0.42) | 30.68 (0.42) | 31.86 (0.31) | 30.98 (0.23) | 35.87 (0.26) |
| 2 | 34.29 (1.05) | 32.91 (0.27) | 33.75 (0.80) | 33.83 (0.36) | 38.27 (1.01) |
| 2 | 33.95 (0.71) | 32.95 (0.23) | 34.11 (0.93) | 33.76 (0.45) | 37.83 (0.08) |
| 2 | 33.71 (0.32) | 33.02 (0.45) | 33.73 (0.41) | 33.41 (0.20) | 38.26 (0.35) |
| 1 | 33.96 (0.16) | 33.44 (0.49) | 33.75 (1.09) | 34.12 (0.41) | 38.08 (0.43) |
| 1 | 33.64 (0.60) | 34.03 (1.84) | 35.42 (0.27) | 35.58 (0.97) | 38.22 (1.94) |
| 1 | 34.17 (0.58) | 34.76 (0.66) | 36.51 (1.27) | 35.29 (0.73) | 40.58 (1.22) |
