# Supplementary material for: Genome-enhanced detection and identification of fungal pathogens responsible for pine and poplar rust diseases
Source: PLoS One. 2019 Feb 6;14(2):e0210952. doi: 10.1371/journal.pone.0210952 (PMC6364900; doi:10.1371/journal.pone.0210952)
Supplement: S7 Table — The replicate numbers in the first column correspond to the three independent extractions from the same number of spores. (DOCX) [file pone.0210952.s008.docx]

**S7 Table. Detection limit of *Melampsora* genus- and *Melampsora medusae*-specific assays, using a known amount of *Melampsora medusae* f. sp. *deltoidae* urediospores from which DNA was extracted.** The replicate numbers in the first column correspond to the three independent extractions from the same number of spores.

| No. of spores  per reaction | Means of C_t_ values calculated from three technical replicates (± SD) | | | | |
| --- | --- | --- | --- | --- | --- |
|  | MEL40 | MEL100 | MEL176 | MM53 | MM74 |
| 30 000 | 20.57 (0.06) | 19.69 (0.11) | 20.01 (0.03) | 19.13 (0.10) | 20.88 (0.02) |
| 30 000 | 20.04 (0.44) | 19.66 (0.04) | 19.80 (0.02) | 18.84 (0.19) | 20.65 (0.10) |
| 30 000 | 20.68 (0.22) | 19.99 (0.04) | 20.29 (0.09) | 19.33 (0.16) | 21.10 (0.08) |
| 500 | 26.00 (0.32) | 25.45 (0.09) | 25.60 (0.09) | 24.89 (0.19) | 26.54 (0.07) |
| 500 | 26.19 (0.20) | 25 47 (0.11) | 25.58 (0.06) | 24.96 (0.15) | 26.70 (0.15) |
| 500 | 26.81 (0.09) | 25.94 (0.02) | 26.01 (0 03) | 25.45 (0.10) | 27.03 (0.05) |
| 100 | 28.31 (0.12) | 27.60 (0.10) | 27.84 (0.20) | 27.02 (0.18) | 28.72 (0.08) |
| 100 | 28.72 (0.06) | 27.94 (0.10) | 28.00 (0.04) | 27.33 (0.02) | 29.08 (0.10) |
| 100 | 28.93 (0.06) | 28.02 (0.10) | 28.00 (0.08) | 27.38 (0.06) | 29.30 (0.17) |
| 25 | 30.87 (0.13) | 30.05 (0.29) | 30.05 (0.05) | 29.51 (0.12) | 31.23 (0.24) |
| 25 | 30.90 (0.22) | 30.30 (0.08) | 30.28 (0.08) | 29.55 (0.00) | 31.31 (0.42) |
| 25 | 30.91 (0.27) | 30.14 (0.14) | 30.19 (0.14) | 29.63 (0.15) | 31.45 (0.13) |
| 10 | 31.96 (0.24) | 31.75 (0.38) | 31.76 (0.16) | 30.67 (0.17) | 32.39 (0.20) |
| 10 | 32.06 (0.31) | 30.96 (0.20) | 31.06 (0.22) | 30.73 (0.09) | 32.21 (0.14) |
| 10 | 32.15 (0.09) | 30.79 (0.21) | 30.80 (0.22) | 30.31 (0.21) | 32.17 (0.22) |
| 2 | 34.86 (0.71) | 33.74 (1.11) | 33.56 (0.88) | 33.46 (0.46) | 35.89 (1.33) |
| 2 | 34.77 (0.81) | 33.96 (0.70) | 33.57 (0.88) | 32.90 (0.12) | 35.28 (0.80) |
| 2 | 34.30 (0.41) | 34.49 (1.40) | 33.21 (0.24) | 32.94 (0.45) | 34.75 (0.80) |
| 1 | 36.46 (1.38) | 34.06 (0.12) | 35.27 (1.04) | 33.31 (0.58) | 35.66 (0.26) |
| 1 | 34.68 (0.52) | 35.39 (1.49) | 35.41 (1.04) | 35.03 (0.64) | 36.31 (0.39) |
| 1 | 38.79 (5.82) | 35.61 (1.57) | 35.01 (0.73) | 35.18 (1.34) | 35.51 (0.28) |
