# Supplementary material for: Genome-enhanced detection and identification of fungal pathogens responsible for pine and poplar rust diseases
Source: PLoS One. 2019 Feb 6;14(2):e0210952. doi: 10.1371/journal.pone.0210952 (PMC6364900; doi:10.1371/journal.pone.0210952)
Supplement: S8 Table — The replicate numbers in the first column correspond to the three independent extractions from the same number of spores. (DOCX) [file pone.0210952.s009.docx]

**S8 Table. Detection limit of *Melampsora* genus- and *Melampsora larici-populina*-specific assays, using a known amount of *Melampsora larici-populina* urediospores from which DNA was extracted.** The replicate numbers in the first column correspond to the three independent extractions from the same number of spores.

| No. of spores  per reaction | Means of C_t_ values calculated from three technical replicates (± SD) | | | | |
| --- | --- | --- | --- | --- | --- |
|  | MEL40 | MEL100 | MEL176 | MLP104 | MLP133 |
| 30 000 | 22.22 (0.06) | 19.94 (0.02) | 20.46 (0.08) | 19.95 (0.08) | 20.36 (0.09) |
| 30 000 | 22.31 (0.09) | 20.20 (0.03) | 20.55 (0.13) | 20.11 (0.08) | 20.57 (0.07) |
| 30 000 | 22.38 (0.10) | 20.44 (0.04) | 20.72 (0.07) | 20.26 (0.10) | 20.81 (0.03) |
| 500 | 28.41 (0.05) | 25.93 (0.14) | 26.65 (0.08) | 26.13 (0.13) | 26.45 (0.08) |
| 500 | 28.99 (0.12) | 26.14 (0.05) | 27.08 (0.14) | 26.46 (0.15) | 26.72 (0.10) |
| 500 | 28.37 (0.10) | 26.12 (0.08) | 26.63 (0.09) | 26.22 (0.04) | 26.64 (0.07) |
| 100 | 30.53 (0.19) | 28.14 (0.10) | 28.96 (0.04) | 28.13 (0.08) | 28.48 (0.10) |
| 100 | 30.55 (0.16) | 28.47 (0.08) | 29.04 (0.07) | 28.42 (0.13) | 29.04 (0.07) |
| 100 | 31.43 (0.05) | 28.22 (0.03) | 29.31 (0.32) | 28.59 (0.21) | 28.85 (0.11) |
| 25 | 32.69 (0.32) | 29.81 (0.16) | 31.38 (0.16) | 30.68 (0.30) | 30.32 (0.11) |
| 25 | 32.20 (0.38) | 29.89 (0.14) | 30.10 (0.11) | 29.83 (0.13) | 30.37 (0.16) |
| 25 | 33.14 (0.53) | 29.88 (0.07) | 32.12 (0.91) | 30.53 (0.13) | 30.46 (0.13) |
| 10 | 34.27 (0.41) | 32.09 (0.20) | 33.59 (0.31) | 32.40 (0.38) | 32.50 (0.11) |
| 10 | 33.71 (0.33) | 31.36 (0.29) | 32.10 (0.25) | 32.19 (0.43) | 31.45 (0.40) |
| 10 | 33.65 (0.44) | n/a | 32.24 (0.89) | 32.19 (0.43) | n/a |
| 2 | 35.74 (0.52) | 33.87 (0.36) | 35.40 (1.37) | 33.56 (0.01) | 34.53 (0.88) |
| 2 | 36.04 (0.30) | 34.12 (1.34) | 34.72 (0.43) | 33.93 (0.61) | 34.25 (0.41) |
| 2 | 35.61 (0.31) | 34.84 (0.78) | 34.73 (0.59) | 33.83 (0.67) | 36.67 (0.56) |
| 1 | 37.66 (1.65) | 34.08 (0.92) | 35.23 (1.52) | 35.69 (0.89) | 34.84 (0.53) |
| 1 | 38.01 (1.00) | 35.62 (1.90) | 36.98 (0.59) | 36.47 (n/a) | 35.42 (1.05) |
| 1 | 41.48 (6.14) | 35.74 (1.13) | 35.33 (0.20) | 36.32 (1.09) | 36.00 (n/a) |
